# Ionotropic Receptors Identified within the Tentacle of the Freshwater Snail *Biomphalaria glabrata*, an Intermediate Host of *Schistosoma mansoni*

**DOI:** 10.1371/journal.pone.0156380

**Published:** 2016-06-02

**Authors:** Di Liang, Tianfang Wang, Bronwyn A. Rotgans, Donald P. McManus, Scott F. Cummins

**Affiliations:** 1 Faculty of Science, Health and Education, University of the Sunshine Coast, Maroochydore, Queensland 4558, Australia; 2 Molecular Parasitology Laboratory, QIMR Berghofer Medical Research Institute, Brisbane, Queensland, 4006, Australia; George Washington University School of Medicine and Health Sciences, UNITED STATES

## Abstract

*Biomphalaria glabrata* (*B*. *glabrata*) is an air-breathing aquatic mollusc found in freshwater habitats across the Western Hemisphere. It is most well-known for its recognized capacity to act as a major intermediate host for *Schistosoma mansoni*, the human blood fluke parasite. Ionotropic receptors (IRs), a variant family of the ionotropic glutamate receptors (iGluR), have an evolutionary ancient function in detecting odors to initiate chemosensory signaling. In this study, we applied an array of methods towards the goal of identifying IR-like family members in *B*. *glabrata*, ultimately revealing two types, the iGluR and IR. Sequence alignment showed that three ligand-binding residues are conserved in most *Biomphalaria* iGluR sequences, while the IRs did exhibit a variable pattern, lacking some or all known glutamate-interactingresidues, supporting their distinct classification from the iGluRs. We show that *B*. *glabrata* contains 7 putative IRs, some of which are expressed within its chemosensory organs. To further investigate a role for the more ancient *IR25a* type in chemoreception, we tested its spatial distribution pattern within the snail cephalic tentacle by *in situ* hybridization. The presence of *IR25a* within presumptive sensory neurons supports a role for this receptor in olfactory processing, contributing to our understanding of the molecular pathways that are involved in *Biomphalaria* olfactory processing.

## Introduction

*Biomphalaria glabrata (B*. *glabrata)* is an air-breathing aquatic pulmonate gastropod mollusc in the family Planorbidae found in freshwater habitats across the Western Hemisphere. It is an intermediate host for the *Schistosoma mansoni* parasite, causing one of the most prevalent parasitic infections in humans, known as schistosomiasis (also called Bilharzia). Schistosomiasis is a crippling disease found in 76 countries (primarily developing countries) [1] and causes approximately 280,000 deaths per annum in sub-Saharan Africa alone [2, 3]. It was believed that preventive praziquantel chemotherapy and molluscicides were the most appropriate means of eradicating this overwhelming disease burden [4]. However, the fact that currently available synthetic molluscicides tend to be generally biocidal, affecting many other animals and/or plants in the snail habitat, along with the threat of praxiquantel drug resistance [5, 6], have spurred recognition of the pressing need for a practical and ideal supplementary approach in addition to chemotherapy.

As an important intermediate snail host that is integral to the transmission of a significant human pathogen, *Biomphalaria* presents itself as a powerful model organism for studying the complexities of host-pathogen interactions. As is the case with most molluscs, *Biomphalaria* snails have virtually no hearing and very limited vision so they obtain the vast majority of their information about the environment by smell. Using this dependence, one may envisage the use of a broad-spectrum of chemical cues to manipulate snail behavior, allowing for development of environment-friendly control strategies. Towards realizing an olfactory-mediated control strategy, it is important to be aware of the molecular biological makeup of the molusc’s olfactory system, including the odor chemosensory receptors.

Ionotropic glutamate receptors (iGluRs) are a conserved family of ligand-gated ion channels widespread across vertebrates [7] as well as invertebrates [8]. Ionotropic receptors (IRs), a variant family of the iGluRs, were identified as a novel group of chemosensory receptors in *Drosophila melanogaster* [9]. They were subsequently identified in several other species [8, 10], demonstrating that IRs had an evolutionary ancient function in detecting odors, likely playing a general role in initiating chemosensory signaling.

By allowing neurons to communicate with each other in the brain in response to external signals, iGluRs function in synaptic transmission as receptors for the excitatory neurotransmitter glutamate and related ligands [11]. Based on their main agonist, traditional iGluRs can be divided into three pharmacologically and molecularly distinct receptor subfamilies: α-amino-3-hydroxy-5- methyl-4-isoxazolepropionic acid (AMPA), kainate and N-methyl-D-aspartate (NMDA). AMPA and kainate receptors are commonly grouped as non-NMDA receptors. Of these, the AMPA receptors are best characterized for their function in mediating the fast excitatory synaptic transmission while kainate receptors play a more subtle, modulatory role in this process [8]. NMDA receptors may have appeared after the non-NMDA receptors, and are well known for their role in synaptic and neuronal plasticity, requiring two agonists (glutamate and glycine) for activation [8]. The idea that iGluRs initiate metabotropic signaling has been proposed for some types of mammals [12–14]. For example, GABA released in the supraoptic nucleus of the hypothalamus is mediated by kainate iGluRs through an ionotropic mode of action [15].

Both iGluRs and IRs universally possess a conserved ligand-gated ion channel domain encoded by Pfam domains PF10613 and PF00060 [16]. The ligand-gated ion channel domain is made up of a combination of three transmembrane (TM) regions, an ion channel pore and a large extracellular domain that contains a ligand binding domain (LBD), whose two half-domains (S1 and S2) combine to constitute a ‘‘Venus flytrap” that encloses glutamate and related agonists [17]. Further, almost all iGluRs contain an extracellular amino-terminal domain (ATD, Pfam domain PF01094) involved in the assembly of subunits into heteromeric complexes, which are discernible only in well-characterized *Drosophila melanogaster* IR25a and IR8a [8], but not in other known IR. In order to respond fast to the binding of extracellular ligands through action potential generation, both iGluRs and IRs depolarize these domains by permitting TM ion conduction [18]. Given that IRs possess a similar structure to iGluRs, it is not surprising that IRs evolved from an animal iGluR ancestor without drastic functional modifications, simply transitting in expression from an interneuron (where it modifies synaptic transmission in response to external amino acid) to a sensory neuron (where it may detect chemical signals from the external molecules). Similar to iGluR, IRs are situated in distal membrane regions of neuronal dendrites, but on cilia instead of post-synaptic membranes [8].

Integrating all publicly available data, we found that there is substantial variation in the size of the IR repertoire throughout Protostomes, from three in *C*. *elegans* to eighty-five in the crustacean *Daphnia pulex* and studies [10, 19] point out IRs as the only known putative chemosensory receptors expressed in crustacean antennules. In the study by Croset et al. [8], the gastropod *Aplysia* expresses ionotropic receptors (IRs) in chemosensory organs. However, the picture is far from complete, as besides *Aplysia*, very little attention has been given to the genetic basis of chemoreception in other molluscs. Whether nor not IRs also play a role in the olfactory system of *Biomphalaria* has remained elusive.

A significant breakthrough will be the annotation of the 931-Mb genome sequence of the *B*. *glabrata*, the third complete molluscan genome available to date after oyster and octopus [20, 21]. Its completion has provided an excellent opportunity to characterize the chemoreceptor repertoire of *Biomphalaria*. In this study, a total of 19 candidate iGluR and 7 IR genes have been obtained by utilizing the resources from this recently available *B*. *glabrata* genome. Of these, 14 sequences are predicted to house a 3TM domain with full-length open reading frames (ORF). We characterized the phylogenetic clustering and carried out an extensive tissue expression profile for all *Biomphalaria* IRs that showed a widespread expression of *BglaIRs* in non-antennal tissues, except for the IR25a which are found predominantly expressed in the tentacle and central nervous system (CNS), implying that in this species the IRs have a more complex function. We also complemented our PCR experiments by analyzing the expression and spatial distribution pattern of IR25a with RNA by situ hybridization.

## Materials and Methods

### Ethics statement

The conduct and procedures involving animal experimentation were approved by the Animal Ethics Committee of the QIMR Berghofer Medical Research Institute (project number P242). This study was performed in accordance with the recommendations in the Guide for the Care and Use of Laboratory Animals of the National Institutes of Health.

### Animal rearing and tissue collection

*B*. *glabrata* (BB02 strain) were maintained in flow-through aquarium tanks at Queensland Institute of Medical Research (QIMR) during January, 2014, in a constant temperature room set to 25°C, and fed to satiety on lettuce. For collection of tissues, animals were removed from the aquarium and relevant tissues dissected out and either (1) embedded in optimal cutting temperature compound for cryostat sectioning to perform *in situ* hybridization, or (2) snap frozen in liquid nitrogen for RNA and protein isolation.

### Gene identification and functional annotation

The *B*. *glabrata* genome and genome protein annotation files were downloaded from the following resources: *Bioinformatics Resource for Invertebrate Vectors of Human Pathogens* (https://www.vectorbase.org/organisms/biomphalaria-glabrata), NCBI contigs file (http://www.ncbi.nlm.nih.gov/Traces/wgs/?val=APKA01#contigs) and NCBI scaffolds file (ftp://ftp.ncbi.nlm.nih.gov/genbank/genomes/ Eukaryotes/ invertebrates/Biomphalaria_glabrata).

To identify target sequences, the *B*. *glabrata* genome was imported into the CLC Genomics Workbench (v6.0; Finlandsgade, Dk). In this framework, previously identified putative *Aplysia* IRs were used to query (tBLASTn and BLASTx) the databases to help guide receptor identification efforts. Annotated genomic regions retrieved from the databases were translated and screened for the presence of recurrent transmembrane motifs using TMHMM Server v2.0 (http://www.cbs.dtu.dk/services/TMHMM/). The proteins that survived this filter were loaded into the Pfam database (http://hmmer.janelia.org/search/phmmer and http://pfam.xfam.org/search) and searched against the set of profile TMHMMs. Multiple sequence alignments for IRs were performed using the Muscle algorithm, with phylogenetic trees constructed using the neighbor-joining method with a minimum 1000 bootstrap replicates for node support. MikTex Texshade software was used to generate multiple sequence alignments and schematics showing amino acid conservation for the presented figures.

*B*. *glabrata* gene nomenclature was based upon a four-letter species abbreviation consisting of an uppercase initial letter of the genus name and three lower case initial letters of the species name (e.g. *Aplysia californica* = Acal; *Biomphalaria glabrata* = Bgla). iGluRs genes were represented according to the subtype of the receptor (GluN for NMDA and iGluR for non-NMDA), and named based on similarities with previously annotated *A*. *californica* iGluRs, or a logical variant where no corresponding gene was identified. An additional number suffix after a point was appended to the ends of these labels, where necessary, to distinguish them from multiple gene models associated with a single contig or scaffold (e.g. BglaGluR8.1).

### Molecular dynamics simulation

The initial conformations of the receptors were built using SWISS-MODEL by sequence alignment with proteins with known 3D structures (template proteins) [22]. The structure with the highest quality estimation (QMEAN score) was chosen, and subjected to the molecular dynamics simulation (MDS) using AMBER version 14 [23]. The structure was imported using the LEAP module of AMBER; the sequence segment(s) that was miss-represented (normally at N- or C- terminus) due to different sequence length of the template proteins, was built as a linear structure using LEAP and linked back to the corresponding positions. The MDS was fully unrestrained and carried out in the canonical ensemble using the SANDER module. The ff14SB force field [24] was employed. Energy minimisation with 2500 steps was first performed to remove unfavourable contacts. The AMBER structure was then heated to 325K over 50 ps to avoid being kinetically trapped in local minima, then subjected to unrestrained MD simulations at 325K for the purpose of peptide equilibration. The structural information was sampled every 1 ps (i.e., 10,000 structures were calculated for 10 ns MD simulation). This MD simulation was continued until the root mean square deviation (RMSD) of structures within a reasonable long time range was stable at/less than 3~4Å. Then, a lowest energy structure was determined, and considered as the representative of the conformations simulated over this period. Visualisation of the systems was effected using VMD software [25].

### Reverse-transcription PCR

Total RNAs were prepared from each tissue [central nervous system (comprising pooled cerebral, pleural, buccal, pedal and abdominal ganglia), tentacle, foot, heart, lung, blood vessel (in close proximity to the heart), gonad, digestive system and cerebral ganglia using Trizol reagent (Invitrogen, CA, USA), and NanoDrop measured the purity and quantity of each RNA sample. First-strand cDNA was generated using random primers and the Superscript Preamplification System for First-strand Synthesis (Invitrogen). PCR amplification was performed using REDTaq DNA polymerase (Sigma) per the manufacturer's instructions with specific primer combinations that were specific to the target gene (**S2 File**). PCR products were visualized by 2.0% agarose gel electrophoresis to confirm transcript expression. Reactions were run in triplicate with tissues obtained from at least five different animals. Controls included no reverse transcriptase. *Biomphalaria* β-actin (487 bp) was used as a control for all cDNA templates.

### *In situ* hybridization

After identification of putative IR genes, one candidate, IR25a, was selected for tissue expression localisation using whole-mount *in situ* hybridisation on *B*. *glabrata* cephalic tentacles. Total RNA was extracted from *B*. *glabrata* cephalic tentacles using TriZol reagent (Life Technologies) following the manufacturer’s instructions. First-strand cDNA was synthesised from 1 μg total RNA using random hexamers and the TaqMan Reverse Transcription kit (Applied Biosystems). A fragment of *IR25a* was amplified from cephalic tentacle cDNA using specific nested primers. The PCR product was then purified using a QIAquick Gel Purification kit (Qiagen) and ligated into a pGEM-T Easy vector (Promega) according to the manufacturer’s instructions, followed by transformation into JM109 competent cells (Promega). Blue-white screening was used to choose colonies for PCR using T7 and SP6 primers (Promega) and plasmid purification was performed using the QIAprep Spin Miniprep kit (Qiagen). Purified plasmid was then amplified using M13 primers before gel purification of bands in the correct size range using the QIAquick Gel Purification kit (Qiagen). Sense and antisense RNA probes were prepared using a Digoxygenin RNA labelling kit (Roche) with T7 and SP6 polymerase. Spatial localization of *IR25a* within *B*. *glabrata* cephalic tentacles was performed essentially as described (Cummins et al., 2009). Colour was developed with NBT/BCIP (Roche) before tissues were cleared in BB:BA for observation. Tissues were photographed using an Olympus BX60 with Nomarski optics and a Nikon Digital Sight DS-U1 camera.

## Results

### Identification of *B*. *glabrata* ionotropic receptors (IRs) and ionotropic glutamate receptors (iGluRs)

*Aplysia* IR sequences were used to mine the *B*. *glabrata* genome, leading to the identification of 40 IR-like sequences. Subsequent filtering removed false positives and a primary cut-off (E-value = 1.0E-40) was selected. These BLAST-based annotations underwent manual inspection of gene structure. In total, 26 IR-like sequences were inspected manually for homology to the target query and the corresponding gene models were edited where necessary (**S1 Table**). Of the 26 IR-like sequences, 19 contained typical motifs for iGluRs while the remaining 7 were designated as IRs (**Table 1**). Twenty-five sequences did span at least two of the three characteristic transmembrane domains, ranging in size from 229 to 1092 amino acids. Unigene reference, length, and the number of predicted transmembrane domains for the final sequence dataset are shown in **Table 1**.

**Table 1:** Candidate IRs and iGluRs identified from the *B*. *glabrata* genome.

Gene name	Length (bp)	ORF (aa)	TMD	Full length	ATD	Ligand binding residues (R,T,D/E)
BglaIR1	873	291	2	No	No	No
BglaIR2	722	229	2	No	No	No
BglaIR3	1554	517	3	Yes	No	No
BglaIR4	864	288	1	No	No	R
BglaIR5	1521	506	3	Yes	No	R
BglaIR8a	1443	480	2	No	No	R, E
BglaIR25a	981	327	3	No	No	R
BglaGluR1	2751	917	3	Yes	Yes	R, T, E
BglaGluR1.1	1359	449	3	No	No	R, T, E
BglaGluR2	1434	478	2	No	No	R, T, D
BglaGluR3	2388	795	3	Yes	Yes	R, T, E
BglaGluR4	1659	552	3	No	No	R, T, D
BglaGluR4.1	1620	540	3	No	No	R, T, E
BglaGluR6	2757	919	3	Yes	Yes	R, T, D
BglaGluR6.1	2511	837	3	Yes	Yes	R, D
BglaGluR7	2694	898	3	Yes	Yes	R, T, E
BglaGluR8	1281	427	3	No	No	R, E
BglaGluR8.1	1191	396	2	No	No	R, T, E
BglaGluR9	2190	730	3	Yes	Yes	R, D
BglaGluR9.1	1305	434	3	No	No	R, E
BglaGluR9.2	2631	877	3	Yes	Yes	R, T, D
BglaGluR10	879	292	2	No	No	R
BglaGluN11	1311	436	3	No	No	R
BglaGluN12	3279	1092	3	Yes	No	R, T, D
BglaGluN13	1968	655	3	No	No	R, T, D
BglaGluN14	2757	888	3	Yes	Yes	R, D

R, arginine; T, threonine; D/E, aspartic acid/glutamic acid

ATD, amino-terminal domain

TMD, transmembrane domain

Given that 11 of the 19 putative iGluRs had at least 67% identity with the corresponding iGluRs of *Aplysia* (*Biomphalaria* orthologues), we therefore name these following their orthologous genes. BglaGluR6.2 was named due to multiple copies of an orthologue of a *B*. *glabrata* gene which exist and its relatively low similarity to AcalGluR6. The four novel NMDA type iGluRs were named BglaGluN11 through BglaGluN14 to avoid confusion with the names of non-NMDA type iGluRs, which number up to BglaGluR10. A previous report by Croset et al. [8] did indicate that iGluRs contain the Pfam domain corresponding to the ATD, similarly observed within most of the novel *Biomphalaria* iGluRs.

Regarding the IRs, two candidate IR subunits correspond to IR8a and IR25a, which share 31% and 56% amino acid identities with the spiny lobster *Panulirus argus* IR8a and 25a, respectively. Also, there exists 29% and 53% identity at the amino acid level to the fruitfly *Drosophila melanogaster*, IR8a and 25a, respectively. Further, BglaIR25a showed a higher amino acid identity (69%) with the *A*. *californica* candidate IR25a sequence, a potential orthologue identified in *B*. *glabrata*. The remaining five putative IRs do not display considerable conservation to any reported IRs, particularly in the key functional domains, but retained their characteristic features, and thus these were named using the Arabic numerals 1–5 based on the order of their identification in the *B*. *glabrata* transcriptomes.

### Molecular phylogeny and structure of *B*. *glabrata* IR and iGluRs

**Fig 1:**
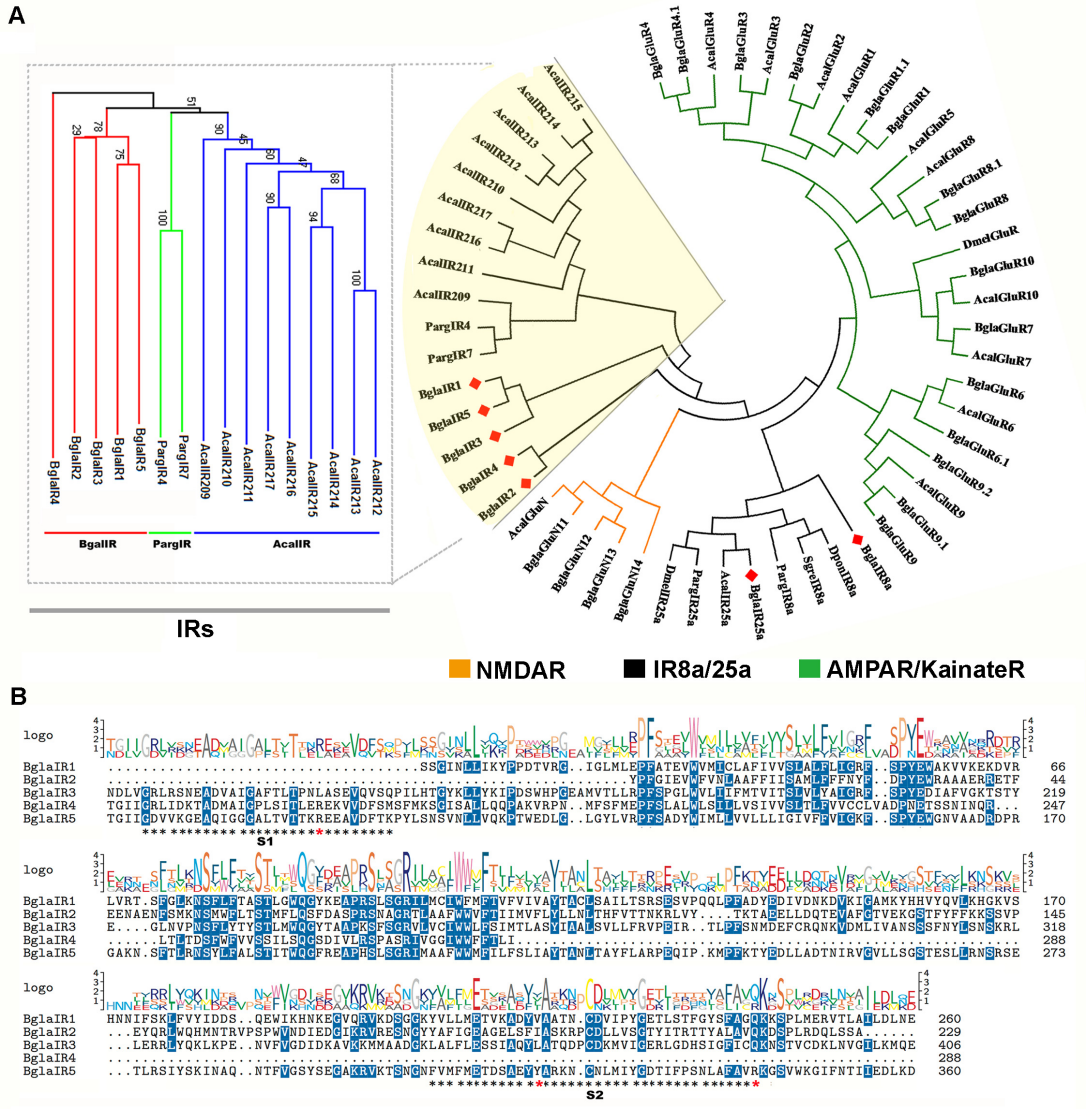
Characterisation of *Biomphalaria glabrata* IRs and iGluRs. (A) Molecular phylogeny for IR and iGluRs from *B*. *blabrata* (*Bgla*), *A*. *californica* (Acal), *S*. *gregaria* (Sgre), *D*. *ponderosae* (Dpon), *P*. *argus* (Parg) and *D*. *melanogaster* (Dmel). Bootstrap supports two IR subfamilies. The 7 newly identified *Biomphalaria* IRs are highlighted with red diamonds. Phylogenetic tree of nonIR8a/25a IRs is shown for 5 *B*. *glabrata*, 2 from *P*. *argus* and 9 from *A*. *californica*. Clades are indicated by different colours. All gene accession numbers can be found in **S2 Table.** (B) Alignment of predicted amino acid sequences of 5 candidate *Biomphalaria* IRs (BglaIR1-5), including regions encoding putative ligand-binding domains; S1 and S2 domains are shown by black asterisks below the sequences. Three key ligand-binding residues (R, T and D/E) are marked with red asterisks. Blue shading indicates identical or similar amino acids. Sequence logo conservation is presented above the sequence.

Comparative alignment between all *B*.*glabrata* iGluRs and IRs was performed (**S1 File**), then aligned with other known and novel candidates and reference sequences of IRs and iGluRs retrieved from NCBI, including *Panulirus argus*, *Aplysia californica*, *Biomphalaria glabrata* and *Drosophila melanogaster* (**Fig 1A)**. It is immediately obvious that there are four primary phylogenetic groupings with the presence of two different phylum-specific IR lineages. All putative *B*. *glabrata* iGluRs have been dispersed over two groups (NMDA and non-NMDA) and clustered with their corresponding orthologous genes into a group, in congruence with the BLAST results. The non-NMDAR (AMPAR/KainateR) group is the largest, containing 11 and 15 members from *A*. *californica* and *B*. *glabrata*, respectively, while the NMDAR group includes AcalGluN and 4 NMDA type iGluRs of *B*. *glabrata*.

The second largest cluster next to the NMDAR group, composed of nine *Aplysia* IRs and two *Panulirus* IRs (IR4 and 7), along with five *B*. *glabrata* IRs (IR1-5), was labeled as an IR group. In addition to the phylogenetic clustering of these sequences into the primary IR group, the Neighbor-joining tree further clustered these IRs into three clear species-specific subfamilies; homology ranged from 19% to 26% between *Panulirus* and *Biomphalaria* IRs and 21% to 35% between *Aplysia* and *Biomphalaria* IRs. Even though they grouped together and largely clustered into a separate monophyletic clade, the five *B*. *glabrata* IRs (includes 3 partial and 2 full-length) exhibited weak similarities when compared together, and the relevant phylogenetic separation is mirrored by noticeable structural differences shown in their alignment analyses (**Fig 1B**).

The newly identified candidate *Biomphalaria* IR25a and 8a formed a distinct cluster together with their counterparts from the other species (two PargIRs, SgreIR8a, DponIR8a, antennal DmelIR25a and IR25a of *A*. *californica*,) and, apart from the existing IR lineages, formed a separated clade next to the existing IR25a/8a lineages. BglaIR25a bears a relatively high similarity to the other broadly expressed olfactory IR25a, regardless whether molluscan (69% with *A*. *californica*) or with non-molluscan (56% with *P*. *argus*, 55% with *M*. *mediator*, 54% with *S*. *gregaria*, 53% with both *D*. *busckii and H*. *assulta*). Although its strongest level of similarity is with PargIR8a, the novel BglaIR8a is only 31% similar to *Panulirus* sequence at the amino acid level. Yet it can be located clearly together with the IR family in the IR25a/8a clade as shown in **Fig 1A.** BglaIR8a shows a less clear relationship with its selected counterparts but appears to fall in the phylogenetic vicinity of the major IR25a/8a probably because of the lack of overall homology (currently there is no IR8a identified in *Aplysia* or any other mollusc).

### Structural features of the ligand-binding domain

**Fig 2:**
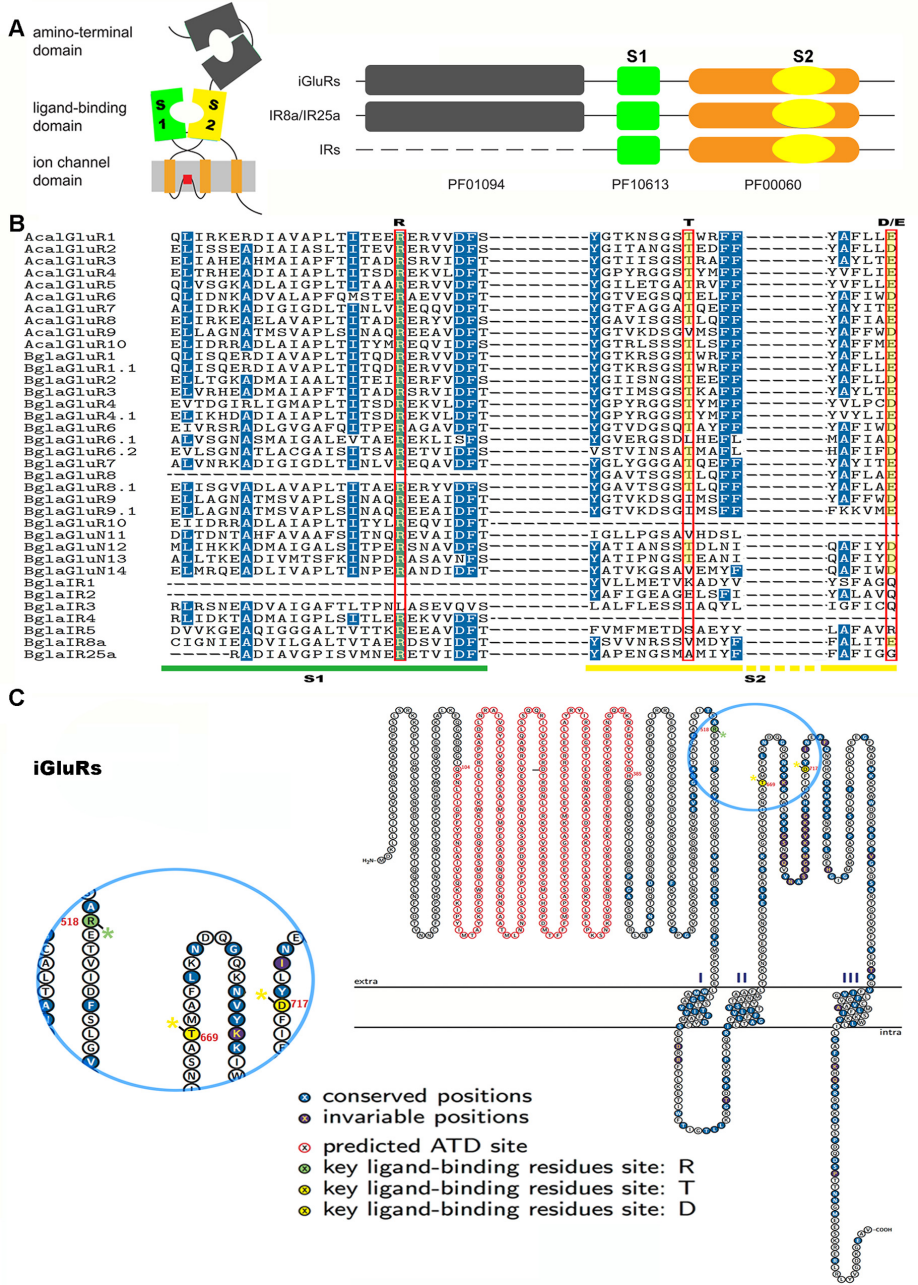
Analysis of ligand-binding domains in *Biomphalaria glabrata* IRs and iGluRs. (A) Left: Protein domain structure of conventional iGluRs/IRs in schematic form [8]. Right: Illustration of the three Pfam domains present in iGluRs and IRs. Both IR8a and IR25a possess the Pfam domain corresponding to the iGluR ATD. All other IRs lack the same homology to the ATD. (B) Alignment of S1 and S2 ligand-binding domains from putative *B*. *glabrata* iGluRs and IRs with *A*.*californica* iGluRs. *Biomphalaria* and *Aplysia* S1 and S2 ligand-binding domains were manually aligned. Blue shading indicates identical or similar amino acids. Three key ligand-binding residues (R, T and D/E) are boxed. S1 and S2 domains are marked with coloured lines at the bottom. (C) Schematic representation of *Biomphalaria* iGluRs, showing conserved and invariable amino acids. Predicted ATD site is highlighted in red and the region of key ligand-binding residues is magnified and shown in yellow and green.

The interaction of glutamate receptors with their ligand is supposed to occur within a “Venus flytrap” that is formed by an extracellular two-lobed ligand-binding domain and three ligand-binding residues (R, arginine; T, threonine; and either D, aspartate or E, glutamate) that align to form salt bridges with the glutamate ligand [17]. **Fig 2A** illustrates the orientation and protein domain structure of conventional iGluRs/IRs and three Pfam domains present in the iGluRs and IRs.

Sequence alignments of the LBD, which is specific to this protein family, based on conserved residues in S1 and S2, were used to help make a final decision with respect to the potential nomenclature of the iGluRs or IRs. As shown in **Fig 2A**, the amino acid sequences of 19 candidate *Biomphalaria* iGluRs and 7 IRs were aligned with *A*. *californica* iGluRs. A conserved amino acid profile in the three key glutamate binding residues was observed in all *Biomphalaria* iGluRs, except in the case of BglaGluR6, 9 and BglaGluN11, 14 which are predicted to contain one or two. However, the profile was not conserved for the candidate *Biomphalaria* IRs, which lack one or more residues, confirming their membership of the IR sub-family rather than the iGluR sub-family. As indicated by the alignment, these IRs contain variable key glutamate binding residues.

Protein structural analysis demonstrated that all candidate *B*. *glabrata* iGluRs contain three key residues at relative positions (**Fig 2B** shows R518, T669 and D717 based on BglaBluR6.2). Additionally, ATD sites are found preceding the LBD S1 domain.

### Tissue-specific expression of *B*. *glabrata* IRs

**Fig 3:**
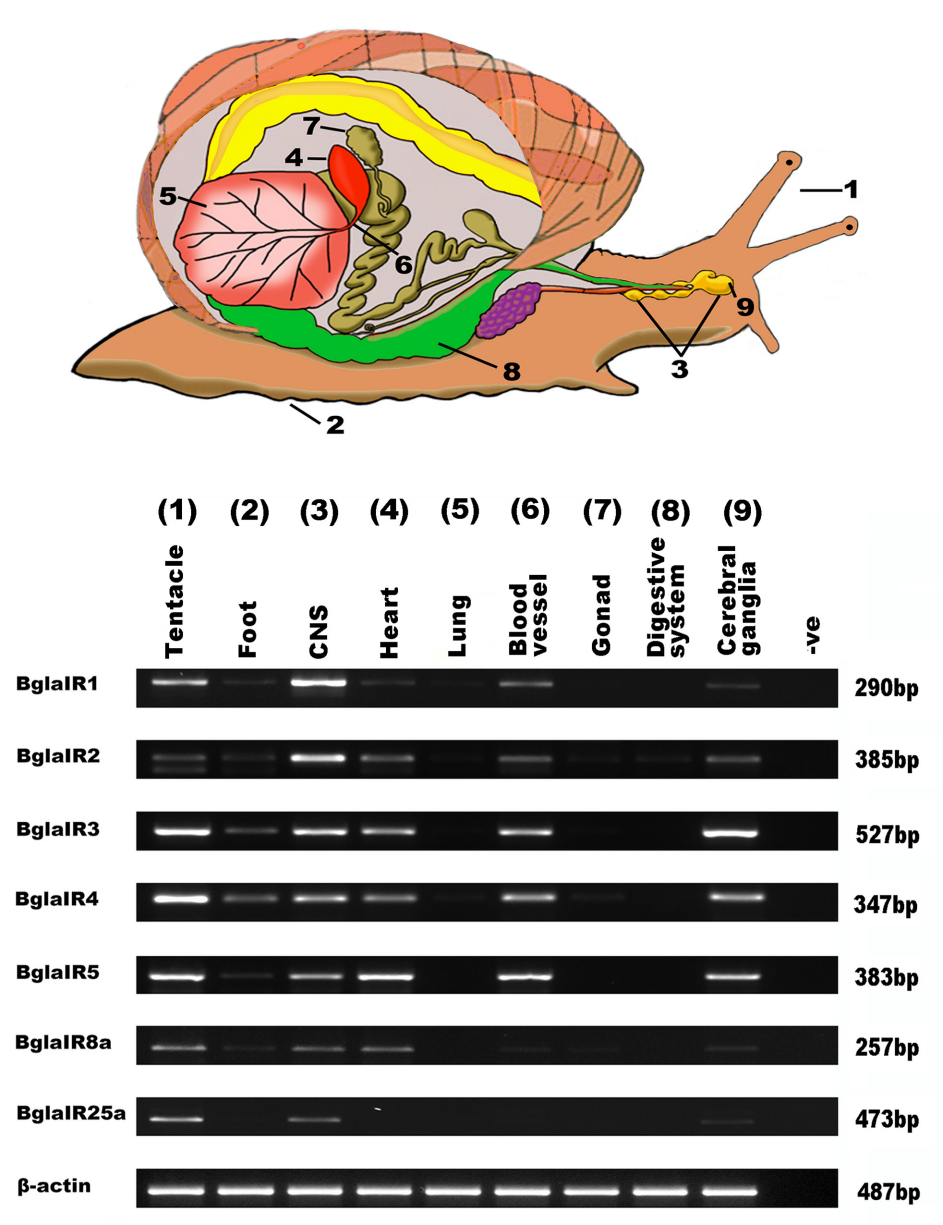
Tissue expression of *Biomphalaria glabrata* IRs. Top: Schematic representation of *B*.*glabrata* showing tissues used for RT-PCR. Bottom: RT-PCR detection of 7 *Biomphalaria* IR genes in different tissues. *Biomphalaria* IRs can be detected in both olfactory and non-olfactory tissues. No expression could be detected from the lung or gonad. No amplification was detected in RNA samples in the absence of reverse transcription (data not shown) or template (-ve). Control RT-PCR products for comparative analysis of gene expression correspond to the β-actin.

Expression profiles of all *BglaIRs* were performed to compare expression in defined tissues with gene-specific primers by RT-PCR as shown in **Fig 3**. Tissues investigated included the sensory regions of adult olfactory organs, as well as non-sensory tissues such as the central nervous system (comprising pooled cerebral, pleural, buccal, pedal and abdominal ganglia), reproductive tissues and various tissues of the visceral mass. All IRs showed widespread expression patterns in tentacle, foot, CNS, cerebral ganglia, and heart and blood vessels, except for *IR25a* that appeared to be exclusive to the tentacle and CNS, including the cerebral ganglia. No expression was detected in lung, gonad and digestive system.

### Characterization and spatial expression pattern of *B*. *glabrata* IR25a

**Fig 4:**
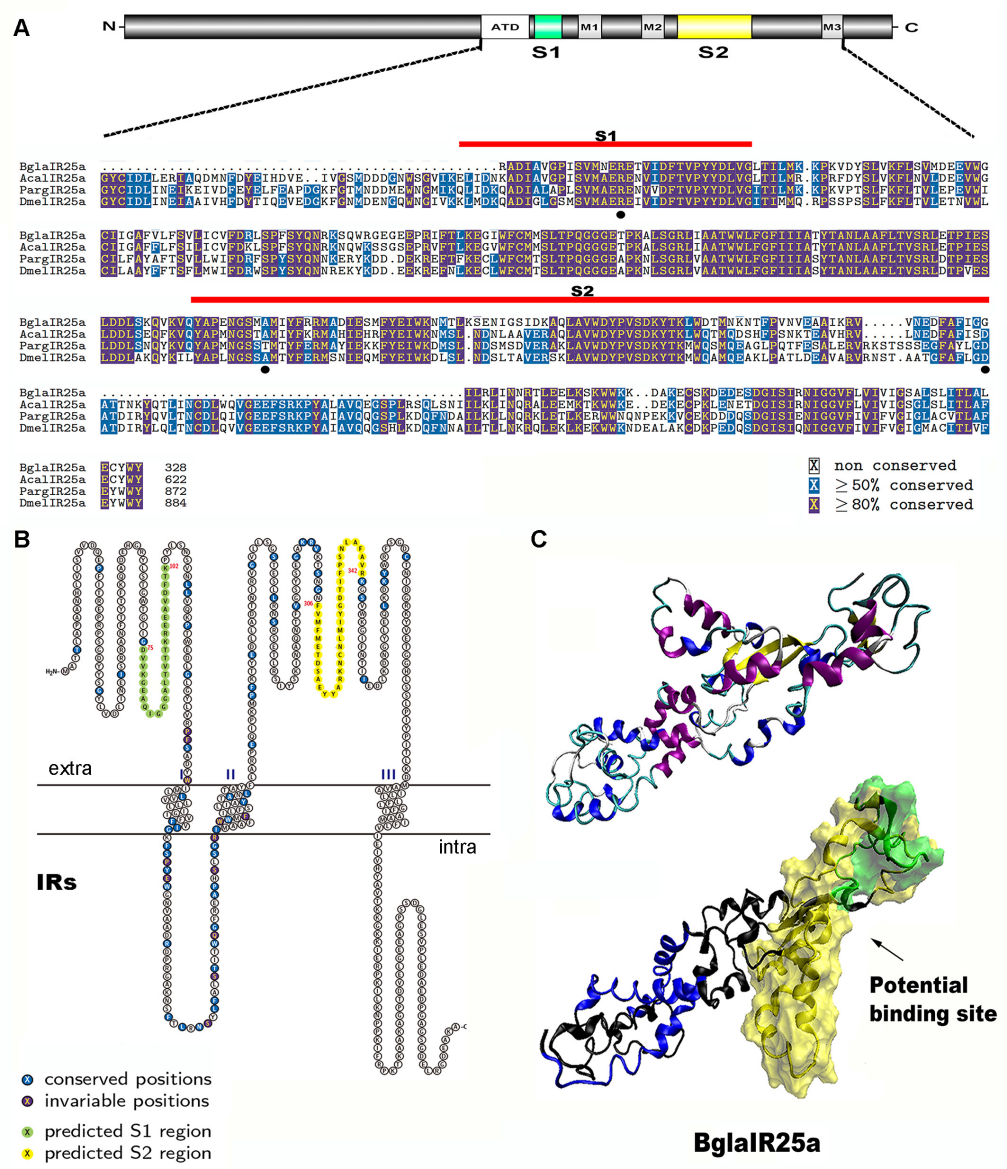
Analysis of *Biomphalaria glabrata* IR25a. (A) The protein domain organization of a typical IR25a is shown above a protein alignment of *Biomphalaria* (Bgla), *Aplysia* (Acal), *Panulirus* (Parg) and *Drosophila* (Dmel) IR25a. Conserved amino acid residues are highlighted in purple (≥80% conserved) and blue (≥50% conserved), and ligand-binding domain S1 and S2 domains are shown with red lines above the sequences. Three key ligand-binding residues (R, T and D/E) are marked with a black dot. (B) Schematic representation of *Biomphalaria* IRs, showing conserved and invariable amino acids. Predicted S1 and S2 region are highlighted in green and yellow, respectively. (C) Structure of BglaIR25a predicted by SWISS-MODEL in conjunction with MDS. Top: tertiary structure, purple-α helix, blue-3-10 helix, yellow-β sheet, cyan-turn and white-random coil. Bottom: space filling of predicted binding site, yellow-predicted ligand binding S1 region, green-predicted ligand binding S2 region, and blue-predicted TM region.

A comparative multiple amino acid sequence alignment of candidate *Biomphalaria* IRs and reference sequences of IR25a retrieved from NCBI, including *P*. *argus*, *A*. *californica* and *D*. *melanogaster* is shown in **Fig 4A**. The five sequences comprised 327 to 947 amino acids so we chose to restrict the character sets to 410 alignable positions, in order to maintain a conservative approach. All the IR25a receptors selected displayed remnants of classical IR motifs at corresponding positions and the predicted domains that are critical structural regions responsible for detecting odorous ligands and contributing to ligand specificity, are highlighted by lines above the alignment. On one hand, the putative glutamate-interacting key residues (R,T,D/E) are completely conserved only in lobster IR25a and two of these three residues are conserved in *Aplysia* and *Drosophila* IR25a. In contrast, with *Biomphalaria*, only one key residue is conserved in the predicted amino acid sequences of IR25a. On the other hand, all these IR25a retain the R residue in S1 that interacts with glutamate in the iGluRs. However, the glutamate binding residues in the S2 sequences are not conserved other than in PargIR25a, suggesting the S2 domain has a much more variable sequence. Furthermore, when considering just the S2, the unequal distribution of variable amino acids and, in particular, their strongest variability in overall length (varies between 84 and 93 amino acids), displays significant variation.

Protein structure analysis and alignment for the *B*. *glabrata* IRs reveals that they share a conserved ligand-gated ion channel structure closely resembling that of conventional iGluRs and IRs with the 2 Pfam domains (PF10613 and PF00060,) formed by an extracellular two-lobed LBD, an ion channel pore and three TM regions. We selected BglaIR5 as a representative to demonstrate the conserved and variable amino acids among these *Biomphalaria* IRs, displaying the predicated ligand-binding S1 domain (D75-K102) and S2 (F306-R342) domain regions (**Fig 4B**). The structure model of the Venus flytrap domain of *Biomphalaria* IR25a is shown in **Fig 4C**, while the respective putative ligand binding site is indicted with an arrow.

**Fig 5:**
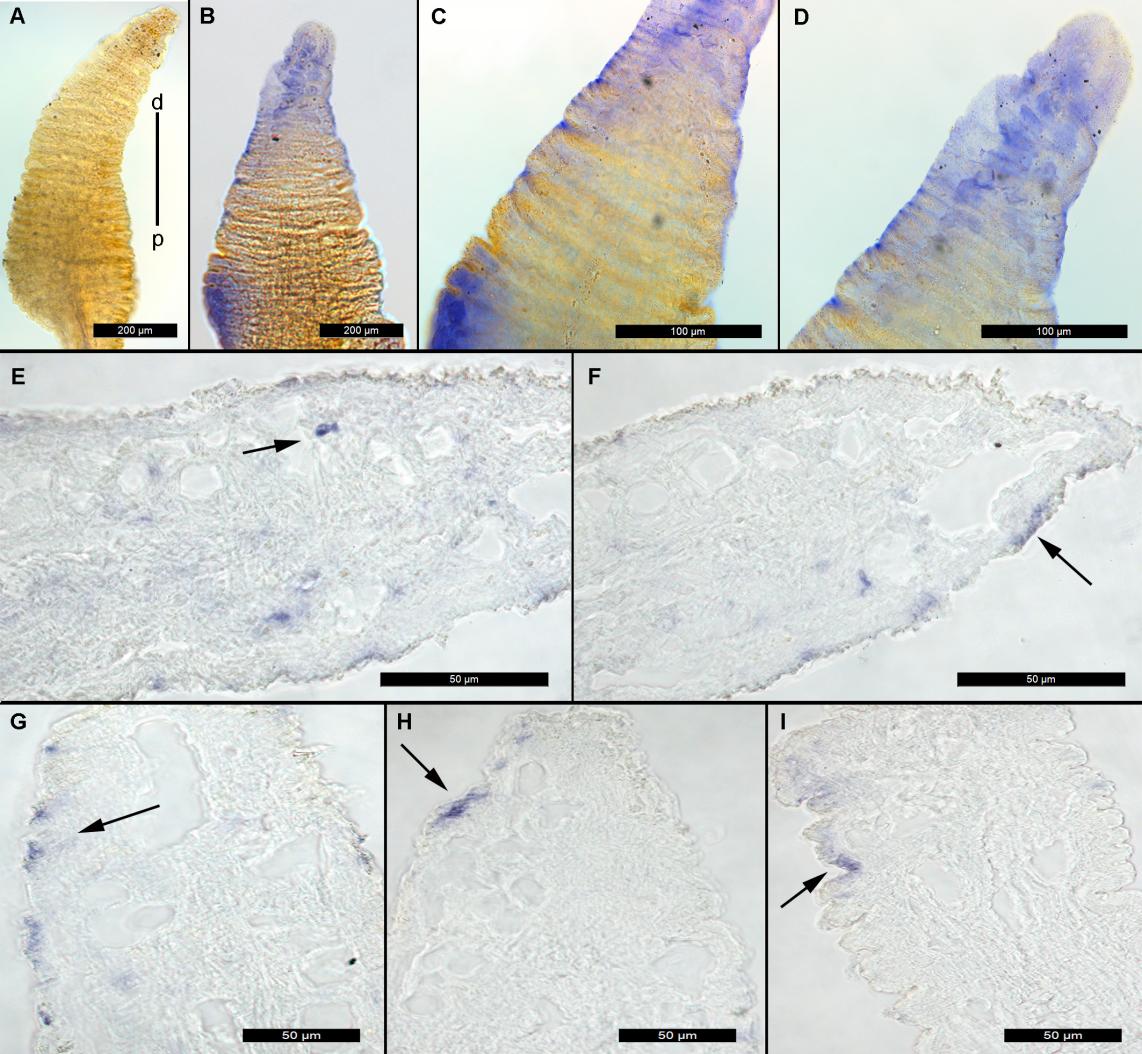
Expression of *BglaIR25a* as detected by *in situ* hybridization in *Biomphalaria glabrata* tentacle. (A) Control whole-mount *in situ* hybridization on tentacle tissue with a DIG-labelled sense riboprobe for *BglaIR25a*. No signal is apparent. (B-D) Whole-mount tentacle probed with antisense riboprobe for *BglaIR25a*. (E-I) Cryostat sections showing cellular localization of *IR25a* within central and peripheral cells (arrows). d, distal; p, proximal.

To further explore the expression pattern of *BglabIR25a* in olfactory tissues, we analyzed its cellular spatial distribution in the *B*. *glabrata* anterior tentacle by whole-mount *in situ* hybridization. No tentacle staining was observed using a sense riboprobe for *BglabIR25a* (**Fig 5A**), while localization was clearly visible within the distal and proximal tentacle regions using an antisense riboprobe (**Fig 5B–5D**). Expression within the epithelium, as well as that of the neuropil was determined following cryostat sectioning (**Fig 5E–5I**). This location is typical of sensory neurons, although we lack an unambiguous neuronal marker to confirm this identification.

## Discussion

The availability of the *B*. *glabrata* whole genome sequence has provided a basis for the *in silico* identification and analysis of undiscovered and novel receptors. Application of the homology-based searches against the original *B*. *glabrata* predicted proteome assemblies led to the identification and examination of 7 putative IRs that possess regions homologous to the LBD site of *Aplysia* IRs, as well as 19 iGluRs.

Identification of receptors was initially focused on the most highly-conserved structural features that extend throughout the iGluR and IR families; then sequences were manually re-annotated to allow for a more refined phylogenetic analysis. For instance, it had been established that the LBD exhibits 3 key residues (R, T, D/E) at fixed positions within S1 and S2, forming a “Venus flytrap” structure [8]. Sequence alignment showed that these highly conserved residues are present in most *Biomphalaria* iGluR sequences, while the IR exhibits a variable pattern, lacking some or all known glutamate-interacting residues, supporting their distinct classification from iGluRs.

Phylogenetic analysis confirmed the division of the 26 receptor sequences into two distinct types (iGluRs and IRs) where 7 novel IR sequences could be categorized into two groups, that is the IR25a/8a and other IRs. Our phylogenetic analysis is congruent with previous findings with respect to the more ancient IR25a/8a lineages neighboring the non-NMDA group. BglabIR1-5 are clearly distinct from the BglabIR25a/8a receptors and show only distant similarity to the *Aplysia* IR sequences, and therefore represent a newly defined IRs cluster, probably reflecting the snail’s very different ecological niche (freshwater versus marine). The lack of obvious orthologs between these two molluscan species suggests expansion or contraction of these receptors occurred after the splitting of the Gastropoda lineage.

A likely role for these BglabIRs as chemosensory receptors may be inferred based on their noted expansion as well as spatial expression that includes the animal’s chemosensory organs by RT-PCR. Furthermore, our whole mount *in situ* hybridization experiments enabled visualization of *IR25a* expression in the tentacle of *Biomphalaria*, including the proximal and distal regions, supporting a functional role in the detection of olfactory stimuli in all regions of the organ. By comparison, in rhinophore sections of *Aplysia dactylomela*, *IR25a* has been demonstrated to be expressed in small clusters of cells of a characteristic neuronal morphology close to the sensory epithelial surface [8]. Also, the *IR25a*-related gene (OET-07) from *Homarus americanus*, the American lobster, is expressed in topographically defined subpopulations of mature olfactory sensory neurons [26]. Similarly, the presence of *IR25a* has been documented in almost all of the lobster antennules, in a similar fashion, specifically within putative chemosensory cells [10, 27, 28]. Together, these results are consistent with at least some of the *Biomphalaria* IRs having a chemosensory function.

Previous studies, coupled with the results presented here confirm that IR25a is likely the oldest IR family member, present in the early protostomian lineage, more than 600 Mya [8, 29]. A representation of the IR25a from 4 species of Protostomia was used to unify protein structure predictions across species, exhibiting a modified LBD. For example, *Biomphalaria* and *Aplysia* receptor sequences show a deletion of 5 amino acid residues in the S2 domain region compared with *Drosophila* and *Panulirus* (**Fig 4**). It is therefore tempting to speculate that the length and structure of the LBD may play a functional role in recognizing various odor ligands.

Our tissue-specific expression studies show that BglaIR25a is exclusive to the tentacle and CNS (of those tissues tested), while other IRs were detected in other tissues, including the muscular foot, which may also contain chemoreceptor cells. IR expression determined in non-sensory tissues supports the idea that IRs may play a more general role in *Biomphalaria* chemosensation, also described in *Aplysia* [8]. The expression of *IR* genes in the blood circulatory system (heart and blood vessel) is particularly intriguing, suggesting a possible role in endocrine-mediated signaling. We anticipate that the knowledge gained from studies on the chemical responses of the foot and blood circulatory system will help us determine whether IRs are possibly involved in sensing or reacting to particular cues in these tissues. This should, in turn, also be informative for determining the role of IR members in the tentacles and CNS of *Biomphalaria*, and to establish the exact cues that each receptor is detecting. Another important point pending further investigations is how the odors transfer to the receptor exposed on the dendrite. In lobsters and other crustaceans, water-soluble odors are believed to dissolve in the mucus covering the aesthetascs and diffuse through the cuticle into the receptor lymph space where they contact the dendrite [30–32]. Whether this principle applies to molluscs or whether a yet unknown mechanism is in operation remains to be tested.

It has been found that both excitatory and inhibitory olfactory signaling in gastropod olfactory sensory neurons are mediated via G-protein-coupled second messenger pathways, which are the largest superfamily of transmembrane proteins involved in cell signaling. For example, water-borne chemical and pheromone detection in *Aplysia* may involve Gaq and can be blocked by antisera specific for phospholipase C (PLC) and Ins(1,4,5)P_3_R [33]. Together with these previous findings, it appears that the GPCRs are not the only known putative chemosensory receptors expressed, which is entirely consistent with the findings of others [8, 34] where chemosensory neurons of *Aplysia* represent an olfactory hybrid and utilize both classes of olfactory receptors. Since GPCRs have been established as key receptors in olfaction for other species, it raises the interesting possibility that IRs may also act in concert with GPCRs in *B*. *glabrata*, where both pathways may contribute to the output of gastropod olfactory sensory neurons. Furthermore, IRs have been detected in the rhinophore and oral tentacle of two *Aplysia* genera, *A*. *dactylomela* and *A*. *californica*, coupled with 19 candidate iGluR and 7 IR genes identified in this study. IR subunits have been found present in the olfactory tissue of two divergent gastropoda subclasses, pulmonates (*Biomphalaria*) and opisthobranchs (*Aplysia*), hinting at a general role of this ion channel family in initiating chemosensory signaling in the Gastropoda. Indeed, we additionally found other IR genes with similarity to *B*. *glabrata* IR25a within publically accessible databases of other aquatic and terrestrial molluscs such as oyster (e.g. *Crassostrea gigas*), *Lottia gigantea* and *Lymnaea stagnalis*. Therefore, our study also allows for a more thorough understanding of evolutionary relationships between the locotrophozoans and the more popular model organisms that belong to other metazoan clades (e.g. the ecdysozoa and deuterostomes).

## Supporting Information

S1 File Comparative alignment between all *Biomphalaria glabrata* iGluRs and IRs. (PDF)

S2 File Gene-specific primers used for RT-PCR of IR genes. IR protein sequence alignments are annotated with primer regions (underlined) and amplified regions highlighted. IR nucleotide sequences have been annotated with primer regions highlighted.
(PDF)

S1 Table Information about all 26 *B*.*glabrata* IR-like sequences. (XLSX)

S2 Table Accession numbers for Fig 1. (XLSX)
