# Supplementary material for: Ionotropic Receptors Identified within the Tentacle of the Freshwater Snail Biomphalaria glabrata, an Intermediate Host of Schistosoma mansoni
Source: PLoS One. 2016 Jun 2;11(6):e0156380. doi: 10.1371/journal.pone.0156380 (PMC4890853; doi:10.1371/journal.pone.0156380)
Supplement: S1 File — (PDF) [file pone.0156380.s001.pdf]

```
BglaGluR1.1  MDLNLDRDFFAQFIYGGVDITAFQFLNNRSET.YRKWQQIWEQ.....YKDQYPDLFPLKTGSALMMDAVLLL.........HKALTSDLQKHKSLKENAVRTCD   90
BglaGluR1    MDLNLDRDFFAQFIYGGVDITAFQFLNNRSET.YRKWQQIWEQ.....YKDQYPDLFPLKTGSALMMDAVLLL.........HKALTSDLQKHKSLKENAVRTCD  346
BglaGluR2    .MKLLVDTGHWFQIDAALAGDAFRLFSSITNT.YFIPDRHSEL..............................................................   41
BglaGluR3    GDLGMLEMNLTNFKIGGVNITGFQIVDPYNTT.SELFLSTWAN.....LDPTYWPGAGSKHVHYEAALAADAVRLFNSAFMSIIKKDPSFLKRMRTTGNTVKCTD  223
BglaGluR4    GIPLIGRFRFIGYCSDLAEMVSRNVGYEYHIR.FVKDGEYGRK..............................................................   50
BglaGluR4.1  .........................................................................................................    0
BglaGluR6.1  RDVVMNVLQAAQQIHLLHDKIDWFVTYPDFED.FLSENTTWSG.TLYGLQLLREEKIPQNISDVVDLHNVTMSRLDLATAIDVVGLLRHILWKEAPGCNRERLLD  332
BglaGluR6    KLISVRNHYFTTFVTVLNPQHTRLVLDEALSLSLFSTPHKWLL.VNLGLREYDLSRFVDSYANVTVIRLMMDYNSMYCKLQHDYINLRRAVFHDAIMLYRQMNGS  369
BglaGluR7    ITLDLGLLDLEDYRYGSANITAYRLIDPARDK.VVNVKTDWLR....HAKKGHESPLMGYAEIEGNSSHTLLTETALAYDAYELFAYALMAYSKVQEVATMNQPC  342
BglaGluR8    .........................................................................................................    0
BglaGluR8.1  .........................................................................................................    0
BglaGluR9.1  .........................................................................................................    0
BglaGluR9.2  RDNLIRKVLVKARSYGMLHQPDMTFFFMDPASYYEPFSGASKM...........YELGLFSRRCELLAYRYIRPEGTTIEENNAAAIDTAKLTSLALDKYLRLPK  339
BglaGluR9    ETVNILLDQARDLAMLSMPPYGWLFYDPGDDI.KILFESYRDISCNFTVLMLLPFNFSLPSGSQSRLNYSLAS...........DALKLVSSAYNIIAENTQASN  212
BglaGluR10   .........................................................................................................    0
BglaGluN11   ............MVVGRSKSVRWSVKVRCSSG.QSNTSVRWSV..............................................................   30
BglaGluN12   ESVDFQTHFSCNVQSQATNQSARSLYWKYGEHLYKKVEQSTSKSTDSRGGRFPLKTSPFSSKRDIDFGFSKSRDIARESRFEGFTRKPLFRDRLSGPLTGPNDLD  200
BglaGluN13   DGEILYRHMLNVSIPGETHLKFNQNGTLKQTD.LIIWNLQWLG........GSKNTTEWKEVGRWTVNGLKMS..................EITWPGGGSTPPSG  150
BglaGluN14   EGRAILSRFQTLAEEVNIRVNAEKIYHDAELMGMTGEDWAWIV......SEQAFEAYNIPVGFLGVHLVNGTNEVNHIKDAVEVIGEAFIRLMSSDNISSPPTGC  231
BglaIR25a    .........................................................................................................    0
BglaIR8a     ...MWINEGFQMVKCPQVQTQGFQVYPSRHVT.........................................................................   29
BglaIR1      .........................................................................................................    0
BglaIR2      .........................................................................................................    0
BglaIR3      .........................................................................................................    0
BglaIR4      .........................................................................................................    0
BglaIR5      .........................................................................................................    0
consensus

BglaGluR1.1  ASSSRYSDGGSSL...................................LSALNG.ETTWQSSD...FQDLTFKPYSP........ETAPHNKTWKVATVIEPPFV  148
BglaGluR1    ASSSRYSDGGSSLLSALNGVQFEGLTGPVVMNNGRRTQYSIDVYKLRFKEPMRK.ETTWQSSD...FQDLTFKPYSP........ETAPHNKTWKVATVIEPPFV  439
BglaGluR2    ..YNNGTKGIQCWRDPPVPWMHGPAIRDALLKHGVRVNTKLDIMHLEYRTTLRS.IGTWEHG....................................VGENPFV  107
BglaGluR3    DSDVKTGHGHVVLEEMKKTKFEGVTGHVAFNEFGQRKEFTLDVYNVAMTRGTAK.IGYWNEKE.........AKFIAQNPRLFQSDPANVNRTRIVTTIIKEPYV  318
BglaGluR4    .......................QKDGTWNGVIGELIKHPLLLLLSSVALGDSY.GHMGDSYGHMGDSYSHMCDSYGHMGDSYGHMGDSYGHMGDSYSHMGDSYG  131
BglaGluR4.1  ....................MDGKSGNINFHATGQRINYSVDVQEVTMYKGISI.LGTWHSRD...GFNVYGEEQVLDKA.....NRTIDNRTRIVTSIDEEPYL   76
BglaGluR6.1  DHIVSAEAFTWQLKNTTSPYNGALGQYIWDKSSKGRTNFTIDILRYIGYTKQEI.GQVVFING...TADVTLSQFNQTRA.VLAEPLEMKKIKLRVATQQQDPFI  432
BglaGluR6    VGGGQKTEKGKMRQTVRRLEMDGCTGHLDFSVQGGQRQESFLQLMTLEGYKTGE..SGTWRSKQSELKQRVEPSRSYSTVSRLE..GNVFGNEPLRVTVMIEDPFV  470
BglaGluR7    DKMYTWRHGNSLLNYMKSSAIEGLSGLIKYDG.GERMDFVLDLLSLTPKGLEV..VGKWDRKH..........RLNITRENVTPTDTKITNKKLIVTTYITKPYV  434
BglaGluR8    .........................................................................................................    0
BglaGluR8.1  .....................................................................................MGSRRPKEIT..........   10
BglaGluR9.1  ..............................................MSLFNELK.RGYWYSEP......................YVMDLQLKFFDIQYAEKPFT   36
BglaGluR9.2  SNKDVIDNEKFLRVIKETNFTDGRTGKIYFDKNGQRKNFHLYLYDHGGEESMYKTIARWGPNG..ANSDLRLNLTNSTQHDNGDREYGIMPDLVRIVVVEEDPFV  442
BglaGluR9    NSLTDISLKFWMNRVLRNSNVTGLTGMFQFNAHGRRNNFYLTLTSFTGVENYL..RGYWYSEP..YVMDLQLKFFDIQYAVNITSPFPLKGRKAQVVMIEEKPFT  313
BglaGluR10   ........................................................................................................M    1
BglaGluN11   ............ARALVRTNMYGLTGQVAFDDDG...FLNISKFHVRNLVYDGR.QSVWQDIGCVQGKEVRPFGIIWPGDAKSLKAETEGKKRYKVVTNPVQPFV  119
BglaGluN12   PDNPDNPEGTENRDLDLNDDPDASSGSSSYKNSSRKYPKKIGNSKMKPYGKNDV.KRRYAEKHRRPRRQLS.DKFSLNILQKNCWQTVSESNIRNVCEQVKDKYG  303
BglaGluN13   KPKRAFLRIATLYEEPYVIYRQPEEEGKCDDKSLPCHIYQRNEKKVLLVETLFF.VGRWTVNG......LKMSEITWPGGGSTPPSGKPKRAFLRIATLYEEPYV  248
BglaGluN14   NESESWSNGDDVYNALKATKIEGETGQVSFNEKGDRVNPMYEIMNINANRRAMS.VGLFGHKDQAIGLRMMGNNLTWPGNVHVKPKGEKISRNLTIVTLVEKPFV  335
BglaIR25a    .........................................................................................................    0
BglaIR8a     ..................................HTEDTHFELYRLNRLANTF..LGNWSESF...GLTLTQQTF..........PATFTDTHLTVTVNVEPPFI   85
BglaIR1      .........................................................................................................    0
BglaIR2      .........................................................................................................    0
BglaIR3      .................................MDTRHWSVLLLHFAALLTEIH.TETSKPD....TPTPPPNTTTTTVTTTTGSSTTAGPKKLIAITLLDPPFV   67
BglaIR4      ......MHGNSLYDALREVSFQGYSGHVLFDIHGRRQNYSIDVLSLTSGSSLRK.VGQWHSLS...GLWLDREEKVKDQV....RPDMRDNRTVIISTLQVPFLM   91
BglaIR5      ..................................................................................MATTLAPAANHLVISVVDQEPFI   23
consensus                                                                                                          *
```

```
BglaGluR1.1    MRIENRNGAPPM..............GPN......................LDLEGYCIDLIEALARSELFE.YQVYLADD..YG...DKNETDGT.WNGIIGQL  210
BglaGluR1      MRIENRNGAPPM..............GPN......................LDLEGYCIDLIEALARSELFE.YQVYLADD..YG...DKNETDGT.WNGIIGQL  501
BglaGluR2      MRKCPPDGTPC...............SGN......................EQFEGYCVDLLKKISVLLKFD.YAIRLVADRNYG...AR.SENGS.WNGMIGEL  169
BglaGluR3      MISKTIRDGTPK..............VGN......................EPVEGFCIDLTKAVAQKVGFD.YIIQFVKDGSYG...AS.FPNGT.WDGIVGEL  381
BglaGluR4      HMGDSYGQM..........................................GDSYSHMCDSYGHMGDSYGHM.GDSYGHMGDSYG...HMGDSYGH.IGDSYGDM  189
BglaGluR4.1    MAALEVTDGIPL..............IGR......................FRFIGYCSDLAEMVSRNVGYE.YHIRFVKDGEYG...RK.QKDGT.WNGVIGEL  139
BglaGluR6.1    MI........................GPD......................NTYSGFSYDLIQKISENTRYE.FELYETEDDDVK...VK.......TNGMVNAL  480
BglaGluR6      SKRTDS.............................................GDFEGFCIDILEEVSRILGFR.YNISKVPDGKYG......SYKTHGWTGMINEI  523
BglaGluR7      MKDNVT.............................................GQYYGFCIDLLNEIAKTRNFT.YEIQIESKVGKR......IGNNNNWDGVMGAL  487
BglaGluR8      .........................................................................................................    0
BglaGluR8.1    .........................................................DMKLANALAKEVGFT.HEFRIVADNAYG...AK.LSNGS.WNGMIGEL   52
BglaGluR9.1    SLKRDHMLLKGN.......................................NRFEGFAVELIADIAEMLDFE.YEIYLVNDGKFG....NKLPNGE.WNGMIGEL   96
BglaGluR9.2    IRRSEPILG..........................................DYYEGFSIDLIKRLAQELKFK.YELYVSPGNVYG...AKNLLTNQ.WDGMVNEV  500
BglaGluR9      SLKRDHMLLKGN.......................................NRFEGFAVELIADIAEMLDFE.YEIYLVNDGKFG....NKLPNGE.WNGMIGEL  373
BglaGluR10     DKENPTPD...........................................ELYEGFCIDLTKELARIVGFN.FRIELVPDGNYG....SPNTQGE.WDGMVREI   57
BglaGluN11     MTEAPHADYGTCLSDTPCLNLTNKVKSQEVVWQAIEAYENGSTAESGLFVIQCCRGLTIDLLNKLASDLDFE.FTLYIVQDETYG...QR.SSNGT.WSGIMRDL  218
BglaGluN12     LYTDLQDMSGNQTLD....................................VCCTGLSIDLLKRLCTMLNFD.VQLTEVADGSYG...SPINNNESDWTGMVGML  368
BglaGluN13     IYRQPEEEGKC...DDKSLPCHIYQRNERKVLLSNETIF............KCCSGLSMDLLKIFSEQLNFD.FEIKEVYDGQWG...L..LVNKT.WNGLVKAL  331
BglaGluN14     EVTPLPKDGVCRPVYPAKHAFPCKNGTQD......................LCCMGYCMDMLAKISKDVQFN.FTIHLSKDGLFGSFERHNSSDKKYWNGMMGEL  417
BglaIR25a      .........................................................................................................    0
BglaIR8a       FRNTSEP............................................GSYYGYSMDVLTEIAKTVGFT.FTVRECDEGGYG.....MLENGI.WNGCIGNI  139
BglaIR1        .................................................................................................MSS.....    3
BglaIR2        .........................................................................................................    0
BglaIR3        MHEDR.......................G......................TRFTGLAIEVFREIVQQTGYDNFDLKLPNDAEKY...NWEESLLQ.INDLVGRL  123
BglaIR4        LRTSPTMDGVPL..............VDN......................NRFEGYSKDLADAISQHLDFQ.YVLKIIENNEHG...RD.LGNGS.WTGIIGRL  154
BglaIR5        TREPRPSG...........................................DYYSGYLVDLINEISRRANFT.YTFKQADE..HG...RY.LSTG..WTGIIGDV   76
consensus                                                             *   **         *             *            * *

BglaGluR1.1    ISQERDIA.....VAPLTITQDRERVVDFTKPFMDT.GISIMIKKPDKTKPG..VFSFMDPLDTRVW..................................LCIA  273
BglaGluR1      ISQERDIA.....VAPLTITQDRERVVDFTKPFMDT.GISIMIKKPDKTKPG..VFSFMDPLDTRVW..................................LCIA  564
BglaGluR2      LTGKADMA.....IAALTITEIRERFVDFSKPFMNL.GTSIMIKKPDKEKGG..VFSFKNPLSDGVW..................................VSIL  232
BglaGluR3      VRHEADMA.....IAPFTITADRSRVIDFTKPFMSL.GISIMIKRPQPAGKH..FFSFMEPLSSEIW..................................MCII  444
BglaGluR4      GDSYGDMGDSYGHMAPLTITSDREKVLDFTKPFMSL.GISLMIKKPVETDPH..VFSFMRPLSQEIW..................................LCTI  257
BglaGluR4.1    IKHDADIA.....IAPLTITSDREKVLDFTKPFMSL.GISLMIKKPVETDPH..VFSFMRPLSQEIW..................................LCTI  202
BglaGluR6.1    VSGNASMA.....IGALEVTAEREKLISFSYTVMSS.QASILIKKA.DSTTN..YFQFLGPFSGELW..................................AMIL  542
BglaGluR6      VRSRADLG.....VGAFQITPERAGAVDFTKPYITK.GTTVVVKRPEHRIW...IFQFLLPLSNVVW..................................SAIF  585
BglaGluR7      VNRKADIG.....IGDLTINLVREQAVDFTKPFLTL.GITILFKKPAPKALN..LFSFLSPLSFDVW..................................VYMI  550
BglaGluR8      .................................MEI.GVTIVIKKPQSQRPG..VFSFMEPLSLEVW..................................VCII   35
BglaGluR8.1    ISGVADLA.....VAPLTITAERERYVDFSKHFMEI.GVTIVIKKPQSQRPG.............VW..................................VCII  104
BglaGluR9.1    LAGNATMS.....VAPLSINAQREEAIDFTKPFKTR.YISVLMKIPTRETS...YFEFLNPLSPVVW..................................ICTM  158
BglaGluR9.2    LSGNATLA.....CGAISITSARETVIDFSLGVVST.GVNILVKRPDEDLT...IFQFMMPFSLELW..................................MAIL  562
BglaGluR9      LAGNATMS.....VAPLSINAQREEAIDFTKPFKTR.YISVLMKIPTRETS...YFEFLNPLSPVVW..................................ICTM  435
BglaGluR10     IDRRADLA.....IAPLTITYLREQVIDFTKPFLNL.GISILFKVPRKEKPG..LFSFLNPLAIEIWLYVIGESEFYTLAIEIWLYVIGESELYTLAMEIWLYVI  154
BglaGluN11     TDNTAHFA.....VAAFSITNQREVAIDFTDPYFFS.GFSIIYSDRTRETS...MLAFLEPFSTKVW..................................FAIL  280
BglaGluN12     IHKKADMA.....IGALSITPERSNAVDFSMPFLQT.GITIIVAIREGAIS...PTAFLEPYDYPSWS.................................LILL  431
BglaGluN13     LTKEADIV.....MTSFKINPDRASAVNFSVPYLET.GIKIIVALRDGAIS...PTAFLEPYDYASWS.................................LILI  394
BglaGluN14     MRQEADLI.....VAPLTINPERANDIDFTKPFKYQ.GLNILVRKTQKDSS...LASFLQPFQDTLW..................................ILVG  479
BglaIR25a      ...RADIA.....VGPISVMNERETVIDFTVPYYDLVGLTILMKKPKVDYS...LVKFLSVMDEEVW..................................GCII   60
BglaIR8a       ...EADVI.....LGALTVTAERDSVIDFTLPYYDFAGIQIITRRQLSNIN...LFYYIDVFSTQAW..................................LSLI  199
BglaIR1        .....................................GINLLIKYPPDTVRG..IGLMLEPFATEVW..................................VMIC   35
BglaIR2        ..........................................................MYPFGIEVW..................................FVNL   13
BglaIR3        RSNEADVA.....IGAFTLTPNLASEVQVSQPILHT.GYKLLYKIPDSWHPGEAMVTLLRPFSPGLW..................................VLII  188
BglaIR4        IDKTADMA.....IGPLSITLEREKVVDFSMSFMKS.GISALLQQPAKVRPN..MFSFMEPFSLALW..................................LSIL  217
BglaIR5        VKGEAQIG.....GGALTVTTKREEAVDFTKPYLSN.SVNLLVQKPTWEDLG..LGYLVRPFSADYW..................................IMLL  139
consensus          **            **   *     **   *    *     * *           *  *     !
```

```
BglaGluR1.1 IGFLAVSFVLYFVGRFSPYEWSVC............EDAKERTATTVFSITNTLWFS..LGALMQQ.........G.SDISPRSISGRVIGSA............  342
BglaGluR1   IGFLAVSFVLYFVGRFSPYEWSVC............EDAKERTATTVFSITNTLWFS..LGALMRQ.........G.SDISPRSISGRVIGSA............  633
BglaGluR2   LGFIGVSTVLYLVGRFSPYEWDTSQP..........RRPPEEEKRPAFNLANTLWFS..LGALMQQ.........G.SDIYPRSISGRIVGSA............  303
BglaGluR3   FAYIGVSVVLFLVSRFSPNEWH..............LSGYQHSVANDFSISNSLWFS..LGAFMQQ.........G.CDISPRSLSGRIVGSV............  511
BglaGluR4   FAFIGVSVVLFLVSRFSSEEW...............QVDSESKLENDFTIGNSLWFS..LGAFMQQ.........G.CDVLPKSVSGRIVTSV............  323
BglaGluR4.1 FAFIGVSVVLFLVSRFSSEEW...............QVDSESKLENDFTIGNSLWFS..LGAFMQQ.........G.CDVLPKSVSGRIVTSV............  268
BglaGluR6.1 LFIMVAGLALYVMSRFDP.....................TQEGNVQRFDLKESLWYS..LNIILQG.........S.TDYSPQTTSMRAIIAF............  602
BglaGluR6   IAFVSTSLMLFAVSRVN......................SDRQAKYAHNLRESFWYI..WGTLLRG.........S.LSGSPHAISSRIVSSA............  644
BglaGluR7   AAYLCVSFMLFVIARFSPYEWCNPHP..........CNPDTDEVENQFSVMNSLWFT..IGSLMQQ.........G.CEIAPRALSTRLVAGM............  621
BglaGluR8   VAYLTVSIGLFLVSRFSPVEWKKVREHQNNIDWTQVRADKDGLYHNDFSLFNSFWFS..MGALMFQ.........G.SDTCPRSISGRIIGGA............  116
BglaGluR8.1 VAYLTVSIGLFLVSRFSPVEWKKVREHQNNIDWTQVRADKDGLYHNDFSL.................................SISGRIIGGA............  164
BglaGluR9.1 CAFVIVSVILYILERVGRSKDTQEVEITERERERRVRRVKREREKSKRGSKRERERRVREERERDK.........SNTDSTPTTIPGRILTSA............  242
BglaGluR9.2 GASTLVTIVFFAMDYCS.........................EEDRRFTLKETIWFT..IGTLLKR.........G.TDFAPVPISQRILTAG............  618
BglaGluR9   CAFVIVSVILYILER.....................VGRSKDTQEVEITVRESFWFI..FGSLLQG.........N.TDSTPTTIPGRILTSA............  495
BglaGluR10  GAYLIVSFTIFTLARFSPYEWYNPHP..........CNPDTDVVENTFNLSNSLWFT..VGTLMQQ.........G.SDINPRAVSTRLVGDIFYFVAFLSPFVA  237
BglaGluN11  VSAHITAVCMALFEWNSPFG................LNPWGRKRNKNYSLASGLTMV..FSVLFGH.........TVKTKSPKAWPSKVMQNF............  346
BglaGluN12  FSVHATGASIFIFEWLSPYGL....................DQGKTSLRVKTCELQI.............................VRFLANI............  475
BglaGluN13  FSVHATGSSILIFEWLSPYGL...............NRGLTHMRDHKFSLFRSFWLI..WAMLFST.........SVQTDSPKGIASRFLANI............  461
BglaGluN14  LSVHVVALVLYLLDRFSPFGRFK.............LAKSDDTEEDALNLSSAMWFS..WGVLLNS.........GIGEGTPRSFSARVLGMV............  548
BglaIR25a   GAFVLFSVLICVFDRLSPFSYQNRKS..........QWRGEGEEPRIFTLKEGIWFC..MMSLTPQ.........G.GGETPKALSGRLIAAT............  131
BglaIR8a    SVVLLTSLLLWVYEKASFHCLKSCTKSA........GDVKADNLGQTFYLGKSPVLAFNNNTFVSKKPMPQERAPG.GGDAPRSVAGRVLVAG............  283
BglaIR1     LAFIVVSLALFLIGRFSPYEWAKVV...........KEKDVRLVRTSFGLKNSFLFT..ASTLGWQ.........G.YKEAPRSLSGRILMCI............  105
BglaIR2     AAFFIISAMLFFFNYFDPYEWRAAAE..........RRETFEENAENFSMKNSMWFL..TSTMFLQ.........S.FDASPRSNAGRTLAAF............   84
BglaIR3     FMTVITSLVLYAIGRFSPYEDI..............AFVGKTSTYEGLNVPNSFLYT..YSTLMWQ.........G.YTAAPKSFSGRVLVCI............  255
BglaIR4     LVSIVVSLTLFVVCCLVAD.................PNETSSNINQRLTLTDSFWFV..VSSILSQ.........G.SDIVLRSPASRIVGGI............  281
BglaIR5     VVLLLIGIVFFVIGKFSPYEWGNVA...........ADRDPRGAKNSFTLRNSYLFA..LSTITWQ.........G.FREAPHSLSGRIMAAF............  209
consensus         *  *    * **                             *      **                   *     *   * *

BglaGluR1.1 ...............WWFFTLIIISSYTANLAAFLTIEKLVV....SISSADDL......VGHPTIKYGTKRSGTSWRFFE........................  398
BglaGluR1   ...............WWFFTLIIISSYTANLAAFLTIEKLVV....SISSADDL......VGHPTIKYGTKRSGTSWRFFEK...STVDKYVTMRNEMLANADEV  710
BglaGluR2   ...............WWFFTLIIISSYTANLAAFLTIERMEV....PINSADDL......AKQTDIRYGIISNGSTEEFFKN...SNVPVYSKMWSFMKDSEPSV  380
BglaGluR3   ...............WWFFTLIIISSYTANLAAFLTVERMLT....PIESAEDL......ARQTEIQYGTIMSGSTKAFFKN...SQFQTYQRMWAYMTSAQPSV  588
BglaGluR4   ...............WWFFTLIIISSYTANLAAFLTTQRMSG....SIRSAEDL......VKQTEIKYGPYRGGSTYMFFN.....................PDV  382
BglaGluR4.1 ...............WWFFTLIIISSYTANLAAFLTTQRMSG....SIRSAEDL......VKQTEIKYGPYRGGSTYMFFNQ...TTVSLYQRMWSFMTS.QPDV  344
BglaGluR6.1 ...............FWFCVLIIEAAYTANLAAHLTLQQIDN....RIKTVHDL......AGQSSLMYGVERGSDLHEFLQN...TKEDPYERLWAFIKLNEDKV  679
BglaGluR6   ...............WWFFCLIVSSIYTANLAAFLTITVGDV....DMNSAADL......ATQNIFDYGTVDGSQTAYFFEH...TKMRHYATMWAYMYSLSPNS  721
BglaGluR7   ...............WWFFTLIMISSYTANLAAFLTVERMVS....DINSADDL......AKQSKIKYGLYGGGATQEFFEK...NQLPLYIGMWEYMNRTKEHN  698
BglaGluR8   ...............WWFFVLIIISSYTANLAAFLTIERMIN....PIESADDL......LNHPSIKYGAVTSGSTLQFFLN...SDVPVYRQMGEYMKT.HSEV  192
BglaGluR8.1 ...............WWFFVLIIISSYTANLAAFLTIERMIN....PIESADDL......LNHPSIKYGAVTSGSTLQFFLN...SDVPVYRQMGEYMKT.HSEV  240
BglaGluR9.1 ...............WWFFALILISSYTANLAAFLTVKKINT....PIKSVTDL......AQQTKIKYGTVKDSGIMSFFKN...TNIEHFAKMWAQMSEIEPGS  319
BglaGluR9.2 ...............FLFFVLITVSTYTANMAAFLTIKNFGE....TVNSFETL......AESKKIGVSTVINSATMAFLKN.DQGQKNVYKKIWSKVLG..SNG  695
BglaGluR9   ...............WWFFALILISSYTANLAAFLTVKKINT....PIKSVTDL......AQQTKIKYGTVKDSGIMSFFKN...TNIEHFAKMWAQMSEIEPGS  572
BglaGluR10  DINPRAVSTRLVGGIWWFFTLIIISSYTANLAAFLTVERMVS....PIESAEDL......AKQTEI.......................................  293
BglaGluN11  ...............WAFAAIFIIASYTANLAAFIAGKHAGI....NYYDIQD.......PRLQDIRIGLLPGSAVHDSLKN...........GGSKLVSVADRY  414
BglaGluN12  ...............WALFAVVFCASYTANLAAFMITKDDYY....DLSGIQDWRLKNPHHRQPPFKYATIANSSTDLNIAK.......NYKAIHSYMKN.....  549
BglaGluN13  ...............WALFALVFLASYTANLAAFMITKEEYY....DLSGIQDYRLQNPYTMNPPFKYATIPNGSTEANIRT...NHIDMYNYMKAYNLP.....  539
BglaGluN14  ...............WAGFAMIIVASYTANLAAFLVLDRPEA....LISGIDDPRLR...NPNEKFKYATVKGSAVEMYFKR.QVELSTMYRNMENQKKYYTAEA  630
BglaIR25a   ...............WWLFGFIIIATYTANLAAFLTVSRLET....PIESLDDL......SKQVKVQYAPENGSMAMIYFRRMADIESMFYE.IWKNMTLKSENI  210
BglaIR8a    ...............FWFFAIIIMSTFTANLAAFLTVSRMGA....VISSLDDL......LDQTDMKYSVVNRSSVMDYFKRMARIESDFYER.WKNMTLDKSDS  362
BglaIR1     ...............WFMFTVFVIVAYTACLSAILTSRSESVPQQLPFADYEDI......VDNKDVKIGAMKYHHVYQVLKHGKVSHNNIFSKLFVYIDD..SQE  187
BglaIR2     ...............WWVFTIIMVFLYLLNLTHFVTTNKRLV....YTKTAEEL......LDQTEVAFGTVEKGSTFYFFKK...SSVPEYQRLWQHMNTRVPSP  161
BglaIR3     ...............WWLFSIMTLASYIAALSVLLFRVPEIR..TLPFSNMDEF......CRQNKVDMLIVANSSSFNYLSN...SKRLLERRLYQKLKP..ENV  332
BglaIR4     ...............WWFFTLI...................................................................................  288
BglaIR5     ...............WWMFILFSLIAYTANLTAYFLARPEQIPKM.PFKTYEDL......LADTNIRVGVLLSGSTESLLRN...SRSETLRSIYSKINA..QNT  287
consensus                  **** **   ***********                **             **
```

```
BglaGluR1.1   ...........VRESKSTYAFLLESAMNTYYGQQEP.CDTMMVGEKLDYKGYGVASTIN.....FPLR   449
BglaGluR1     LFSEYHEGVKKVRESKSTYAFLLESAMNTYYGQQEP.CDTMMVGEKLDYKGYGVASTIN.....FPLR   772
BglaGluR2     FVSSTNEGIQRVRDSKGKYAFLLDSTFNEYHNQRKP.CNTMKVGRDLDVKGYGIATPRG.....SDLR   442
BglaGluR3     FVRTHEEGIQRVRQSNGKYAYLTESLTIDYVSNRKP.CDTLKVGNNLNSDGFGIGTPLG.....SDLR   650
BglaGluR4     FVKDNDEGIKRVRESNGQYVLP...............CDTMKAGNNLDSKGYGIATPMG.....SDIR   430
BglaGluR4.1   FVKDNDEGIKRVRESNGQYVYLIESTLNEYYSSRLP.CDTMKAGNNLDSKGYGIATPMG.....SDIR   406
BglaGluR6.1   LLDNTTEIINRVKA..GTMAFIADGVTNGYYANQH..CGIESIVQNFQTKDFSLGFPKG.....APYL   738
BglaGluR6     MVRNVDKGFARVNQ..GGYAFIWDSPVIRHKISND..CMLMEIGTPFDLKGYGFAYTKN.....APYG   780
BglaGluR7     FVKSLDEAIQKVKK..GDYAYITESTTAEFVIHRH..CDLMKVGGLLDSKGYGFATPTG.....SPWR   757
BglaGluR8     LVQSNEEGIERVLKSKGKYAFLAESSFIEYINERSP.CDTVSVGGKLNNIGFGVATPRN.....SPLR   254
BglaGluR8.1   LVQSNEEGIERVLKSKGKYAFLAESSFIEYINERSP.CDTVSVGGKLNNIGFGVATPRN.....SPLR   302
BglaGluR9.1   MVDNTTEGFKKVME............................IGPYFDPKGFGIGVPPG.....ATYK   354
BglaGluR9.2   QVVNASEGMKKVKK..GTHAFIFDYLINEAFQNRH..CDVMAVSNPILLQEHGIGMMAG.....APFK   754
BglaGluR9     MVDNTTEGFKKIKT..DDYAFFWDTTVNKFQTLID..CEVMEIGPYFDPKGFGIGVPPG.....ATYK   631
BglaGluR10    ....................................................................   293
BglaGluN11    PVPKTDEGIDWVMP...........................DISLKLSP...................   436
BglaGluN12    YPQTSVKDAIRALKNQEIQAFIYDSTTLEYEFGKDVGCKLKTVGKRIAETGYGLAFPKK.....SQWV   612
BglaGluN13    ...DVESGITALKQGK.IQAFIYDSSVLEYRASRDPKCGLVTVGNRYAMTGYGVGFPPNNKQFKNPWI   603
BglaGluN14    AIADIRLG........ELQAFIWDSPRLEYEASND..CDLTTAGDLFGRSGLGIGLPKN.....SPWT   683
BglaIR25a     GSIDKAQLAVWDYPVSDKYTKLWDTMNKNTFP.....VNVEAAIKRVVNEDFAFIG............   261
BglaIR8a      TNQDSLAVWEYPLETLSDLDLASVSLRNSHFTPTHW.LEAVQHFEALTPNCNKYG.........TIWA   420
BglaIR1       WIKHNKEGVQRVKDSGGKYVLLMETVKADYVAATN..CDVIPYGETLSTFGYSFAGQKK.....SPLM   248
BglaIR2       WVNDIEDGIKRVRESNGYYAFIGEAGELSFIASKRP.CDLLVSGTYITRTTYALAVQKD.....SPLR   223
BglaIR3       FVGDIDKAVKKMMAADGKLALFLESSIAQYLATQDP.CDKMVIGERLGDHSIGFICQKN.....STVC   394
BglaIR4       ....................................................................   288
BglaIR5       FVGSYSEGAKRVKTSNGNFVMFMETDSAEYYARKN..CNLMIYGDTIFPSNLAFAVRKG.....SVWK   348
consensus            *   *                         *     *        *
```
