# Supplementary material for: Ionotropic Receptors Identified within the Tentacle of the Freshwater Snail Biomphalaria glabrata, an Intermediate Host of Schistosoma mansoni
Source: PLoS One. 2016 Jun 2;11(6):e0156380. doi: 10.1371/journal.pone.0156380 (PMC4890853; doi:10.1371/journal.pone.0156380)
Supplement: S2 File — IR protein sequence alignments are annotated with primer regions (underlined) and amplified regions highlighted. IR nucleotide sequences have been annotated with primer regions highlighted. (PDF) [file pone.0156380.s002.pdf]

Gene ID	Forward	Reverse	bp
BglaIR25a	ATCATCGCAACCTACACC	CCGTCACTCTCATCCTCA	473
BglaIR8a	GGCTGGATTTTGGTTCTT	TGTCTTGGTTAGTGCTGT	257
BglaIR1	GAGGTATGGGTTATGATTTG	AGTATAGCACTAAGACAGG	290
BglaIR2	CGTTCTGGTGGGTCTTCA	GTGGTGCGGGTGATATAG	385
BglaIR3	ACTCTACTTCGACCTTTCTCT	CCATCAGCAGCCATCATC	527
BglaIR4	CACTGGAGAGAGAGAAAG	AAACCACCAAATACCACC	347
BglaIR5	CTGGTGGATGTTTATTCTG	GATTGGATGGGAAGATGG	383
β-actin	GGTGTGATGGTTGGTATGG	CTGTTGTGGTGAAGCTGT	487

```
BglaIR25a   .ADIAVGPISVMNERETVIDFTVPYYDLVGLTILMKKPKVDYS...LVKFLSVMDEEVWGCIIGAFVLFSVLICVFDRL..SPFSYQNRKSQWRG..........    90
BglaIR8a    EADVILGALTVTAERDSVIDFTLPYYDFAGIQIITRRQLSNIN...LFYYIDVFSTQAWLSLISVVLLTSLLLWVYEK...ASFHCLKSCTKSAGDVKADNLGQT   238
BglaIR1     ..........................S.SGINLLIKYPPDTVRG..IGLMLEPFATEVWVMICLAFIVVSLALFLIGRF..SPYEWAKVVKEKDV..........    65
BglaIR2     ...................................................YPFGIEVWFVNLAAFFIISAMLFFFNYF..DPYEWRAAAERRET..........    43
BglaIR3     EADVAIGAFTLTPNLASEVQVSQPILH.TGYKLLYKIPDSWHPGEAMVTLLRPFSPGLWVLIIFMTVITSLVLYAIGRF..SPYEDIAFVGKTST..........   218
BglaIR4     TADMAIGPLSITLEREKVVDFSMSFMK.SGISALLQQPAKVRPN..MFSFMEPFSLALWLSILLVSIVVSLTLFVVCCLVADPNETSSNINQR..........   247
BglaIR5     EAQIGGGALTVTTKREEAVDFTKPYLS.NSVNLLVQKPTWEDLG..LGYLVRPFSADYWIMLLVVLLLIGIVFFVIGKF..SPYEWGNVAADRDP..........   169
consensus    **   *    *  *    **  *     *   *   *              ***   !  *       ** ***   *  ****

BglaIR25a   .EGEEPRIFTLKEGIWFCMMSLTPQ...GGGETPKALSGRLIAATWWLFGFIIIATYTANLAAFLTVSRLETP....IESLDDLSKQVKVQYAPENGSMAMIYFR   187
BglaIR8a    FYLGKSPVLAFNNNTFVSKKPMPQERAPGGGDAPRSVAGRVLVAGFWFFAIIIMSTFTANLAAFLTVSRMGAV....ISSLDDLLDQTDMKYSVVNRSSVMDYFK   339
BglaIR1     ..RLVRTSFGLKNSFLFTASTLGWQ...GYKEAPRSLSGRILMCIWFMFTVFVIVAYTACLSAILTSRSESVPQQLPFADYEDIVDNKDVKIGAMKYHHVYQVLK   165
BglaIR2     .FEENAENFSMKNSMWFLTSTMFLQ...SFDASPRSNAGRTLAAFWWVFTIIMVFLYLLNLTHFVTTNKRLVY....TKTAEELLDQTEVAFGTVEKGSTFYFFK   140
BglaIR3     .YEG....LNVPNSFLYTYSTLMWQ...GYTAAPKSFSGRVLVCIWWLFSIMTLASYIAALSVLLFRVPEIRT..LPFSNMDEFCRQNKVDMLIVANSSSFNYLS   313
BglaIR4     ........LTLTDSFWFVVSSILSQ...GSDIVLRSPASRIVGGIWWFFTLI.....................................................   288
BglaIR5     ..RGAKNSFTLRNSYLFALSTITWQ...GFREAPHSLSGRIMAAFWWMFILFSLIAYTANLTAYFLARPEQIP.KMPFKTYEDLLADTNIRVGVLLSGSTESLLR   268
consensus           * * **  *  **   *   *   **** **! * * ** !  *    ***** * **                **  *  *        *

BglaIR25a   R.MADIESMFYEIWKNMTLK..SENIGSIDKAQLAVWDYPVSDKYTKLWDTMN....KNTFPVNVEAAIKRVVNEDFAFIG...........GILRLINNRTLE   273
BglaIR8a    R.MARIESDFYERWKNMTLD..KSD..STNQDSLAVWEYP..LETLSDLDLASVSLRNSHFTPTHWLEAVQHFEALTPNCNKYGTIWATIQDNGLVNSIEDGLEK   437
BglaIR1     HGKVSHNNIFSKLFVYIDDS..QEWIKHNKEGVQRVKDSG..GKYVLLMETVKADYVAATN.CDVIPYGETLSTFGYSFAGQKKSPLMERVTLAILDLNEDMYIQ   265
BglaIR2     K...SSVPEYQRLWQHMNTRVPSPWVNDIEDGIKRVRESN..GYYAFIGEAGELSFIASKRPCDLLVSGTYITRTTYALAVQKDSPLRDQLSSA...........   229
BglaIR3     N...SKRLLERRLYQKLKPE..NVFVGDIDKAVKKMMAAD..GKLALFLESSIAQYLATQDPCDKMVIGERLGDHSIGFICQKNSTVCDKLNVGILKMQEDEKID   411
BglaIR4     .........................................................................................................   288
BglaIR5     N...SRSETLRSIYSKINAQ..NTFVGSYSEGAKRVKTSN..GNFVMFMETDSAEYYARKN.CNLMIYGDTIFPSNLAFAVRKGSVWKGIFNTIIEDLKDNGYLE   365
consensus       *                              *      *      *       *    *     *         *   * *         *

BglaIR25a   ELKSKWWKKDAKECSKDEDESD....................GISIRNIGGVFLVIVIGSALSLITLALECYWY   327
BglaIR8a    VLNENFA.......LITESPLI....QYYT..GQNC........LLTAIGGN......GRADHKPRNSFE....   480
BglaIR1     ELIAKYIHKKQVCPAYDKTKLV....PQKR............................................   291
BglaIR2     ..........................................................................   229
BglaIR3     VLKKKYF..QGGCLAGKGRSYIFEGLPFFDTFGGEPDAIMPMSITITRFSSAFIILTLGIVIAGVLLVIEIYWS   483
BglaIR4     ..........................................................................   288
BglaIR5     QLKDKYWRFSGDCTNIDGRKYV........ETGGHLSSLPIYPITLKDMAVAILLLFLGFIAAMIFLVIEIVHY   431
consensus    *  *                                                     *          *
```

>BglaIR25a

AGGGCAGATATTGCCGTTGGACCCATCTCTGTCATGAATGAAAGAGAAACGGTGATTGACTTTACTGTGCCT
TACTACGACTTGGTGGGACTGACCATCCTGATGAAGAAACCCAAAGTGGACTACTCACTGGTCAAATTTCTC
AGCGTTATGGATGAAGAAGTCTGGGGGTGCATCATTGGAGCGTTCGTCCTGTTCAGCGTTTTAATTTGTGTG
TTTGACAGATTGAGTCCATTCAGTTATCAAAACCGGAAGTCCCAGTGGCGAGGTGAAGGGGAGGAGCCTCGT
ATATTTACACTGAAGGAAGGAATTTGGTTTTGTATGATGTCTTTAACCCCACAAGGTGGCGGGGAGACGCCC
AAGGCTCTCTCTGGTAGGCTGATAGCTGCCACCTGGTGGCTATTTGGTTTCATTATCATCGCAACCTACACCG
CCAACCTTGCGGCTTTCTTGACAGTCTCTAGGCTAGAGACTCCCATTGAGTCATTGGATGACCTGTCAAAACA
GGTCAAAGTTCAATACGCCCCTGAGAACGGGAGTATGGCCATGATTTATTTCAGGAGAATGGCAGATATAGA
AAGTATGTTCTATGAGATTTGGAAGAATATGACCTTGAAAAGTGAAAACATCGGGTCCATAGACAAGGCGCA
GCTTGCTGTCTGGGACTACCCAGTGAGCGACAAGTACACTAAGCTATGGGACACCATGAACAAAAACACATT
TCCGGTCAACGTCGAGGCAGCCATTAAACGTGTGGTCAATGAAGACTTCGCATTTATAGGTGGGATTCTGAG
ACTAATAAACAATCGTACTCTGGAGGAGCTCAAATCAAAATGGTGGAAGAAAGACGCCAAGGAGTGCTCCAA
AGATGAGGATGAGAGTGACGGCATTAGCATCAGAAACATTGGGGGAGTGTTTCTAGTCATTGTCATAGGATC
TGCTCTCTCGTTGATAACCTTGGCACTTGAGTGTTACTGGTAC

>BglaIR8a
ATGTGGATCAATGAAGGATTTCAAATGGTTAAATGTCCTCAGGTTCAAACTCAAGGATTCCAGGTCTATCCG
TCACGTCACGTGACTCACACTGAAGACACCCACTTTGAACTGTACAGGTTGAATCGTCTTGCCAACACCTTTC
TTGGCAATTGGTCAGAAAGTTTTGGATTGACCTTGACCCAGCAAACTTTTCCAGCGACCTTCACAGACACAC
ATCTTACAGTAACCGTTAATGTTGAGCCTCCGTTTATTTTTCGCAATACCAGTGAGCCTGGCTCCTACTACGG
CTACAGTATGGATGTACTCACGGAGATTGCCAAGACAGTTGGATTCACATTTACCGTCAGGGAGTGTGATGA
AGGCGGCTATGGGATGCTTGAGAACGGAATCTGGAATGGATGTATTGGAAATATCGAGGCCGACGTGATCCT
CGGAGCCCTGACGGTGACAGCCGAACGCGACAGCGTCATAGACTTCACGCTGCCCTATTACGACTTCGCTGGG
ATCCAGATAATTACACGGCGACAACTGTCCAACATTAATCTCTTCTATTACATCGATGTCTTCTCAACTCAGG
CCTGGCTGAGTCTAATCTCTGTCGTACTACTTACAAGTCTTCTGCTCTGGGTTTACGAGAAAGCGTCTTTCCA
CTGCCTGAAGTCATGCACCAAAAGTGCAGGGGACGTCAAGGCCGATAACTTAGGTCAAACTTTCTACCTTGG
CAAGAGCCCCGTCCTGGCCTTCAACAACAACACATTTGTCTCCAAGAAACCAATGCCCCAGGAGAGGGCGCCG
GGGGGAGGCGATGCTCCACGCTCTGTCGCTGGCAGGGTTCTCGTGGCTGGATTTTGGTTCTTTGCGATCATTA
TTATGTCCACATTTACAGCCAATTTGGCCGCCTTCTTGACAGTGTCCAGGATGGGAGCGGTCATCTCAAGCCT
GGACGACCTGCTAGACCAAACGGATATGAAGTACTCAGTGGTCAACAGGTCTAGCGTCATGGACTACTTCAA
AAGAATGGCTCGTATTGAAAGTGACTTTTATGAGCGATGGAAGAACATGACTTTAGATAAATCTGACAGCAC
TAACCAAGACAGTCTGGCTGTGTGGGAATACCCGCTGGAAACACTGTCTGACCTTGACCTTGCCAGCGTCAGC
TTAAGGAATTCTCATTTCACTCCAACTCATTGGCTTGAGGCAGTACAGCATTTTGAAGCCCTAACGCCCAACT
GTAACAAGTACGGAACTATCTGGGCTACTATTCAAGACAATGGACTGGTCAATTCAATAGAGGACGGCTTAG
AAAAAGTGCTGAATGAAAATTTCGCCCTCATCACGGAGTCCCCTCTGATACAGTACTACACTGGGCAGAACT
GTCTCCTCACCGCCATAGGGGGAAACGGAGAGCTGATCATAAACCCAGAAACTCCTTTGAATAG

>BglaIR1
ATGTCAAGTGGCATTAATTTGCTTATTAAGTATCCCCCTGACACTGTGAGAGGCATTGGACTGATGCTTGAG
CCTTTTGCAACAGAGGTATGGGTTATGATTTGTCTTGCTTTCATAGTTGTCAGCCTTGCCTTGTTTCTCATTG
GGCGTTTCAGTCCTTATGAATGGGCAAAAGTAGTGAAGGAAAAAGATGTCAGACTAGTTCGAACAAGTTTTG
GTTTGAAGAATAGTTTTCTTTTTACTGCTAGCACCCTGGGTTGGCAAGGATACAAAGAAGCTCCACGTTCAC
TCTCTGGAAGAATACTGATGTGTATCTGGTTCATGTTTACAGTATTTGTCATTGTGGCTTACACTGCCTGTC
TTAGTGCTATACTGACTAGCAGAAGTGAATCGGTTCCACAGCAGTTACCTTTTGCTGACTATGAAGACATTG
TAGACAACAAGGACGTCAAAATTGGGGCCATGAAATACCATCATGTTTACCAAGTCCTAAAACATGGAAAAG
TTTCTCACAATAATATTTTCTCTAAACTCTTTGTGTACATTGATGATTCCCAAGAGTGGATTAAACACAACA
AAGAAGGTGTTCAGCGTGTGAAAGATTCTGGAGGTAAATATGTTTTATTGATGGAGACAGTCAAAGCTGATT
ATGTTGCTGCTACTAATTGTGATGTAATACCTTATGGAGAAACACTTTCTACCTTTGGGTACTCTTTTGCTG
GTCAAAAAAAATCACCTTTAATGGAAAGAGTGACATTAGCCATCCTTGACCTAAATGAAGACATGTACATCC
AGGAGCTGATTGCTAAATACATTCATAAAAAACAAGTTTGCCCAGCTTATGATAAAACCAAACTTGTCCCAC
AGAAGCGA

>BglaIR2
CCCAACTACGTCCAGCATAATCCTTTCCGCATCATGTACCCATTCGGTATCGAGGTGTGGTTCGTCAATCTGG
CCGCTTTCTTCATCATCAGTGCCATGCTGTTCTTCTTCAACTACTTCGATCCTTACGAGTGGCGCGCCGCTGC
AGAACGGCGTGAGACCTTCGAAGAGAACGCCGAGAACTTCAGCATGAAGAATTCAATGTGGTTCCTGACCTC
TACCATGTTCCTGCAGAGCTTCGATGCTTCTCCCCGCTCCAACGCTGGTCGTACTCTGGCAGCGTTCTGGTGG
GTCTTCACCATCATCATGGTGTTCCTGTACCTGCTGAACCTCACCCACTTTGTTACCACCAACAAGAGGTTGG
TCTACACCAAAACCGCAGAAGAGCTCCTAGACCAGACTGAGGTTGCCTTCGGTACAGTTGAAAAGGGCTCCA
CATTTTACTTCTTCAAGAAGTCTAGCGTACCAGAGTACCAGCGTCTGTGGCAGCACATGAACACGCGTGTTCC
TTCCCCGTGGGTCAATGACATCGAAGACGGTATCAAACGAGTACGTGAGAGCAACGGTTACTATGCCTTCAT
TGGTGAAGCAGGCGAGTTGAGCTTCATAGCTAGCAAGAGACCCTGTGACTTGCTAGTGTCTGGCACCTATAT
CACCCGCACCACGTACGCTTTAGCCGTGCAGAAAGATTCTCCGCTTCGTGACCAGTTGTCAAGTGCCAT

>BglaIR3
ATGGACACTCGGCACTGGTCAGTCCTGCTGCTGCACTTTGCAGCGTTACTAACAGAGATTCATACTGAGACTT
CCAAACCAGACACTCCTACACCCCCTCCAAATACAACAACTACAACTGTCACAACAACCACTGGGTCTAGTAC
AACAGCTGGACCTAAGAAGTTGATAGCCATCACTTTACTGGATCCTCCATTTGTGATGCATGAGGACAGAGG

AACACGTTTCACAGGATTGGCCATAGAAGTCTTTCGGGAAATCGTCCAACAGACTGGCTACGATAACTTTGA
CCTCAAGCTACCTAACGATGCTGAGAAATATAACTGGGAGGAGTCTCTGCTACAAATAAATGACCTTGTAGG
CAGATTACGATCCAATGAAGCAGATGTGGCCATTGGAGCCTTCACTCTAACACCTAATCTGGCGTCTGAGGT
ACAAGTCAGTCAGCCCATTCTCCACACTGGCTACAAGCTTTTGTACAAAATACCAGACTCATGGCACCCAGGG
GAGGCAATGGTGACTCTACTTCGACCTTTCTCTCCTGGACTTTGGGTTCTCATCATCTTCATGACAGTCATCA
CCAGTTTAGTTCTCTATGCCATCGGCAGGTTCAGCCCGTACGAGGACATAGCTTTTGTGGGCAAGACCTCAAC
ATATGAAGGCTTGAATGTACCCAATTCATTTTTATACACATACAGCACACTGATGTGGCAAGGTTACACTGC
TGCTCCGAAGTCTTTCTCTGGGAGAGTGTTAGTTTGTATCTGGTGGCTGTTCTCCATCATGACCTTAGCTTCC
TACATCGCTGCTCTTAGTGTCCTGCTTTTTAGGGTCCCTGAAATTCGAACTTTGCCGTTTTCAAATATGGACG
AATTTTGTCGTCAAAACAAAGTGGATATGTTGATTGTAGCCAATTCTTCTAGCTTCAACTACTTGAGCAATT
CCAAAAGGTTATTGGAGCGTAGACTTTATCAAAAGCTGAAACCTGAAAATGTCTTTGTGGGAGATATAGACA
AAGCTGTTAAAAAGATGATGGCTGCTGATGGGAAATTAGCTCTTTTCCTGGAGTCATCTATCGCTCAGTACC
TGGCCACCCAAGATCCGTGTGACAAAATGGTGATTGGTGAAAGACTCGGTGACCATTCCATCGGCTTTATTT
GTCAGAAGAACTCAACTGTTTGTGATAAATTGAATGTAGGCATTTTGAAAATGCAGGAAGATGAAAAAATA
GATGTGTTAAAAAAGAAATACTTTCAAGGTGGGTGCTTGGCAGGCAAAGGCAGAAGTTACATCTTTGAGGGT
CTTCCATTTTTTGATACATTTGGTGGAGAGCCCGATGCCATCATGCCCATGTCTATCACCATCACCAGATTTT
CATCAGCTTTCATCATTTTGACATTGGGGATTGTAATAGCAGGGGTCCTCCTGGTCATTGAAATTTACTGGT
CCAAAAAAAGAGGTTCTCCGGTTCCTCAAAGAATCAATCGAGGAGGTATTGATGATGATACTGAGAGAATAA
GAAATGAGTACCGTGATGAGGTGGAGAGGGCATAA

>BglaIR4
ATGCATGGCAACAGTTTGTATGATGCATTGAGAGAGGTTAGTTTTCAAGGTTATAGCGGCCACGTACTATTT
GACATTCATGGCAGACGCCAAAATTACAGTATAGATGTTTTGTCCTTGACGTCAGGATCAAGTCTTAGAAAG
GTTGGTCAATGGCATTCATTGTCTGGTTTATGGTTAGACAGAGAGGAAAAAGTAAAAGATCAAGTGAGACCT
GACATGAGAGACAACAGAACAGTTATAATCTCCACACTTCAAGTGCCTTTTCTGATGCTTAGGACAAGCCCC
ACAATGGATGGAGTGCCTTTAGTAGATAATAACAGATTTGAAGGTTATTCAAAGGATCTTGCAGATGCAATT
AGCCAACACTTAGATTTCCAGTATGTCTTAAAAATCATTGAGAATAATGAACATGGCAGAGATCTAGGGAAT
GGCTCATGGACTGGAATTATTGGCAGATTAATTGACAAGACTGCAGATATGGCTATAGGACCATTAAGTATC
ACACTGGAGAGAGAGAAAGTGGTGGATTTTTCCATGTCTTTCATGAAGAGTGGCATCTCTGCATTGCTGCAA
CAGCCTGCCAAAGTGAGGCCAAACATGTTCTCATTCATGGAACCCTTCTCTCTAGCCCTATGGCTGAGTATCC
TGTTGGTTAGCATTGTTGTCAGTCTGACTCTATTTGTAGTCTGTTGTCTTGTGGCTGACCCTAATGAGACTTC
AAGTAACATTAATCAGAGACTAACTTTAACAGATTCATTTTGGTTTGTTGTCAGCTCAATATTATCACAAGG
CAGTGACATTGTCTTGAGGTCACCAGCTAGCCGTATTGTCGGTGGTATTTGGTGGTTTTCACTTTGATC

>BglaIR5
ATGGCGACCACCCTAGCCCCAGCAGCCAACCACCTGGTCATCAGTGTGGTTGACCAGGAACCTTTCATCACAA
GAGAGCCAAGACCCAGTGGTGACTATTACAGTGGCTACTTGGTAGATCTAATTAATGAGATCAGCCGAAGGG
CCAACTTCACCTACACATTTAAACAAGCCGATGAGCATGGGCGATATCTGTCCACTGGATGGACGGGGATCA
TCGGTGATGTAGTTAAGGGGGAAGCTCAGATCGGTGGTGGGGCCCTGACCGTGACCACCAAGCGTGAGGAGG
CTGTAGACTTTACTAAACCTTACCTCTCCAACAGTGTGAACCTGCTGGTTCAAAAACCAACATGGGAAGACCT
TGGCCTTGGCTACCTGGTTCGACCATTCTCAGCAGATTATTGGATTATGCTTCTGGTGGTCTTGTTGCTCATC
GGCATTGTCTTCTTTGTCATTGGTAAGTTTAGTCCTTACGAATGGGGCAATGTGGCGGCAGACCGAGATCCC
AGAGGCGCCAAGAACAGCTTCACACTAAGAAACAGTTACCTGTTCGCTCTTAGCACGATTACCTGGCAAGGT
TTCAGAGAGGCACCCCACTCCCTGTCAGGACGTATCATGGCTGCCTTCTGGTGGATGTTTATTCTGTTCAGCC
TAATCGCCTACACCGCCAACCTGACGGCGTACTTCCTGGCTAGGCCTGAACAAATCCCCAAGATGCCGTTTAA
AACTTATGAGGACCTCCTTGCAGACACCAACATACGTGTTGGTGTGTTACTGTCTGGATCTACAGAGAGTCT
GTTGAGAAACAGCCGCTCTGAGACTCTGAGATCTATCTACAGCAAGATCAACGCACAGAACACATTTGTTGG
CAGCTACAGTGAGGGTGCTAAACGGGTCAAGACTTCCAATGGTAACTTCGTAATGTTTATGGAAACAGACAG
CGCCGAGTATTACGCCAGGAAGAACTGCAACCTGATGATCTACGGAGATACCATCTTCCCATCCAATCTGGCC
TTTGCTGTCAGGAAAGGTTCCGTCTGGAAGGGTATTTTCAATACTATCATCGAGGACCTCAAGGACAATGGC
TACCTGGAACAACTTAAGGACAAATACTGGCGCTTTTCTGGCGACTGTACCAACATTGATGGACGCAAGTAT
GTTGAGACAGGTGGACACCTCAGCTCTCTGCCCATCTATCCGATTACTCTGAAGGACATGGCTGTGGCAATCC
TGCTGCTCTTCCTTGGTTTCATTGCTGCAATGATCTTCCTGGTCATCGAGATAGTCCACTATGCGGTCACCAA

GAAGGGCAAGAAGATTGAAAGGCCTAAGATTCTGAAGAACCCACCCAAGATCTTCAGACCCAAGACCAAGGC
GGCCAAAGCTGGACCCACCGACGTAGAGCTTGGAGAAGAAGCAGGGCCCTCTTCTGACGGCCTTGAGAGCGT
CCCCTTGGAGGATGCTGAGGACCCCGGAGCCGGAAGTGGAGATGAGCTTCGTGGTGACGAGGCCAAGGCTTA
A
